# An integrated approach of Ecological Footprint (EF) and Analytical Hierarchy Process (AHP) in human ecology: A base for planning toward sustainability

**DOI:** 10.1371/journal.pone.0250167

**Published:** 2021-04-16

**Authors:** Mahsa Fatemi, Kurosh Rezaei-Moghaddam, Ezatollah Karami, Dariush Hayati, Mathis Wackernagel

**Affiliations:** 1 Department of Agricultural Extension and Education, School of Agriculture, Shiraz University, Shiraz, Iran; 2 Global Footprint Network, Oakland, California, United States of America; Szechenyi Istvan University, HUNGARY

## Abstract

Environmental challenges to natural resources have been attributed to human behavior and traditional agricultural production techniques. Natural resource degradation in agriculture has always been a prime concern in agro ecological research and sustainability analysis. There are many techniques for assessing environmental performance; one of which, ecological footprint (EF), assesses human pressure on the environment and natural resources. The main purpose of this study was calculation of ecological indices including biocapacity (BC) and EF of rural areas of Fars province of Iran. The study was accomplished using survey and structured interviews consisting of three main questionnaires in two different steps. Different agricultural stakeholders, including farmers (for the first step) as well as the policymakers, extension managers and authorities (for the second step) were interviewed. Based on multi-stage stratified random sampling, 50 villages and 423 farmers were selected. Face validity and reliability of the questionnaires were assessed by a panel of specialists as well as conducting a pilot study, respectively. The paradigmatic perspectives of agricultural policy makers and managers (22 individuals) were also analyzed using another specific questionnaire by Analytical Hierarchy Process (AHP). Findings revealed that most of the studied villages faced a critical environmental condition due to the results of ecological indicator which was calculated in the study. According to the four main components of human ecology (POET model) including *Population*, *Organization*, *Environment and Technology*, village groups that differed in terms of sustainability level also showed significantly differences due to population, social participation, use of green technologies and attitude towards diverse environmental management paradigms. The causal model also revealed that population, green technology, social participation and attitude toward frontier economics, which were in accordance with the elements of human ecology model, were the main factors affecting the ecological index. Finally, AHP results determined the dominant economic perspectives of agricultural authorities. A paradigm shift toward the comprehensive paradigm of eco-development plus consideration of the results of the ecological indicator calculation as the base of agricultural planning at the local level were recommended.

## 1. Introduction

All countries face difficult economic and environmental trade-off issues associated with agriculture in rural areas [1]. Concerns regarding agricultural management intensified with the emphasis on productivity growth from the early 1970s due to its dramatic effects on environmental quality and rural vitality [2]. Recent years have witnessed a proliferation of research on the impacts, tradeoffs, and ramifications of agricultural management of rural areas relative to the set of social and ecological goods and services that society demands from landscapes [3, 4]. Much of this work has highlighted the scale and severity of agricultural impacts on ecological systems, as well as the challenge of designing management paradigms and strategies to meet ecosystem services in the context of limited resources and widespread ecosystem degradation [5]. A parallel stream of work has elaborated a variety of landscape analysis, planning and management paradigms and strategies to address some of these challenges [6, 7].

Environmental challenges have almost been rooted in human behaviors and activities, and the behaviors also have been framed by attitudes of each individual. There are different paradigmatic perspectives in terms of environmental management which could be the basis of people`s decisions. The paradigm of frontier economics prevailed in most countries. This paradigm’s concerns about natural resource exhaustion are hard to rationalize from the point of view of economics. Hence, in theory and in practice, the economy became disembodied from nature. In this “man over nature” worldview, nature has been perceived as a body of resources and natural forces which can be channeled and reshaped by science and technology to provide economic growth [8, 9]. The rationale is reflected in the organization of activities established to manage nature. Nature is separated from human experience so that human beings are able to exploit it without limit and consequence. Humans can manage it, use it as a resource or degrade it without fearing the after-effects [10]. Relying on the idea of nature as resource means that nature’s primary purpose is to service unfettered economic growth [11].

Based on this paradigm, many nations, including low income ones, changed over the past 50 years, and have experienced economic growth, a reduction of poverty and improved welfare [12]. These positive aspects, however, have often been accompanied by a corresponding rise in environmental pressures [13]. Humankind has threatened both the planet and its own prospects of survival [14, 15]. Human activities are generating wastes, such as CO_2_, faster than the biosphere’s ability to dispose of them [16]. As a result, the capacity of natural ecosystems to provide the necessary life support systems for humankind is likely to decrease in the next decades [14, 17, 18]. Average per capita consumption of ecosystem goods and services has increased in the last 50 years, leading to a continuous increase in the human Ecological Footprint (EF) [19]. The amount of biological capacity available per person has fallen, as population growth outpaces increases in ecosystem production and yields throughout the world [14, 19]. This has led to growing ecological deficits for nations around the world.

Alternative pro-environmental paradigms have been developed to mitigate the environmental crisis and degradations. It is well recognized that natural resource and environmental issues occur at the intersection of complex natural and social systems [20]. Despite this recognition, conventional paradigms to environmental management continue to follow disciplinary lines to address challenges. Solving environmental problems more effectively requires increased integration of social and natural sciences, novel governance approaches, and a new culture for environmental stewardship. An articulated framework is needed to engender such characteristics into an environmental management approach [21].

In contrast of *frontier economics* (radical economic perspective), the paradigm of *deep ecology* (radical environmental perspective) is an attempt to synthesize many old and some new philosophical attitudes about the relationship between nature and human socioeconomic activity, with particular emphasis on ethical, social, and spiritual aspects that have been downplayed in the dominant economic, mechanistic worldview [22]. Deep ecology fundamentally rejects the dualistic view of humans and nature as separate and different [9]. This worldview sees nature as worth preserving regardless of the economic benefits [23]. Another paradigm, which is called *eco-development* (intermediate perspective), aims to synthesize the areas of overlap and create a new vision, a new philosophy for the development of human societies. Eco-development recognizes the need for economic as well as the need for that growth to be of a qualitatively very different nature from that which has been pursued in conventional economic development [24, 25]. Eco-development sees most development activity as a form of management of the fundamental relationship between society and nature. The use of “development” connotes an explicit reorientation and upgrading of the level of integration of social, ecological and economic concerns. The core of the eco-development paradigm is to restructure the relationship between society and nature into a “positive sum game” by reorganizing human activities so as to be synergetic with ecosystem processes and services [8].

Overall, the paradigmatic viewpoints of policy-makers as well as key authorities would have effect on different policies, programs and activities. Thus, one of the main purpose of the current study was to measure, compare and discuss the paradigmatic perspectives of top agricultural managers and key policy makers involved in agricultural extension in terms of agricultural environmental management. Achieving this goal, AHP technique was accomplished. The AHP, is one of the most commonly applied multi-criteria decision making techniques which is useful when there are different alternatives or indicators in decision making. It is a powerful and comprehensive methodology, based on pair-wise comparisons, designed to facilitate sound decision making using both empirical data as well as subjective judgments of the decision maker [26].

### 1.1. Ecological footprint: Definition and background

Environmental degradation and depletion of natural resources have been attributed to human behavior and traditional agricultural production techniques which refer to human ecology science. Human ecology can be defined as the study of structure and change in sustenance organizations or resource groups which support human populations within dynamic and constraining environments [27]. Natural resource degradation in agriculture has always been a prime concern in agro ecological research and sustainability analysis. Measuring the extent of environmental degradation in agriculture is therefore essential for countries dependent on agriculture [28]. The search for indicators of sustainability is a recurrent theme in the literature on environmental science, and environmental management and policy. Scientists and practitioners have struggled with selecting indicator components, aggregation procedures, and weights. There are many techniques for assessing environmental performance which can be applied to a farm. Formal environmental management systems such as ISO14001 and techniques such as Life Cycle Assessment [29], Environmental Impact Assessment [30] and EF [31–33] could all be applied to agricultural processes. Indicators and indices have a growing importance in environmental assessment, monitoring and sustainable development issues. Indicators and indices can be used for a wide variety of purposes, such as to assess current conditions, predict trends, compare situations, evaluate policy implementation and monitor ecological degradation [34].

In last two decades, the EF has been introduced as an ecological indicator to assess human pressure on the environment and natural resources. Introduced by Rees [35] and developed by Rees and Wackernagel [36] as a doctoral dissertation at University of British Colombia, Canada, EF and BC of 13 countries were calculated and reported. Afterwards, the trend of Ecological Footprint Accounting (EFA) has been continued by inauguration of an international network called Global Footprint Network (GFN) assessed the EFs and BCs of the world as well as all countries separately and published annually. The EF is a resource and emission accounting tool designed to track human demand on the biosphere’s regenerative capacity [37]. By tracking a wide range of human activities, the EF monitors the combined impact of anthropogenic pressures that are more typically evaluated independently (CO_2_ emissions, fish consumption, land-use change, etc.) and can thus be used to understand the environmental consequences of the pressures humans place on the biosphere and its composing ecosystems [38]. The EF measures the amount of biologically productive land and water area an individual, a city, a country, a region, or all of humanity uses to produce the resources it consumes and to absorb the waste it generates under current technology and resource management practices. This demand on the biosphere can be compared to Biocapacity (BC), a measure of the amount of biologically productive land and water available for human use. Biologically productive land includes areas such as cropland, forest, and fishing grounds, and excludes deserts, glaciers, and the open ocean [39]. In other words, while the EF shows the demand on nature, the BC tracks the supply side of the equation [40, 41]. The EF and BC are both measured in standard units called global hectares (gha). One gha represents a standardized hectare with world average productivity [42, 43]. EFA has several advantages [44]: it is scientifically robust, widely used for territorial and production analysis, and easily understandable by non-experts. EFA can be used at several geographical scales: from world and nations [45] to regions or villages [46].

Decision makers have become increasingly interested in understanding the impact of current life-style and consumption patterns upon global ecosystems [47]. This change in policy focus has brought about an increased interest in the use of footprint indicators for policy assessment. In contrast to the territorial, production-based approach used for international agreements such as the Kyoto Protocol and adopted by most environmental statistics, footprint indicators account for impacts induced by consumption, including production as well as trade flows [48]. EFA has been many applications in different agricultural aspects. This ecological index was assessed in a study for eight fruit productions of orchards in Northern Italy by Cerutti et al. [44]. Findings revealed that hazelnut, kiwifruit and pear had higher EF_product_ and got the first to the third rank among all other fruits. Thus, these applicable results could be helpful for policy-makers in order to recommend special cultivation patterns to the farmers. Similar study evaluated sustainability of swine manure fertilization in orchard through EFA, results showed that almost the same EF for the three manure fertilized systems (liquid slurry, covered slurry and solid fraction) with average of 0.96 gha and the highest EF can be found in the mineral nutrition system (1.14 gha) [49]. Another study was calculated the EF of three methods of agriculture including conventional, organic and integrated. The results indicated that conventional agriculture had the higher EF and reversely the organic had the lowest [43]. Wang et al. [50] calculated and analyzed the EF of Taiwan from 1994 to 2007. The per capita ecological footprint of Taiwan was 5.09 gha in 1994, and increased to 6.54 gha in 2007. Furthermore, the per capita ecological deficit in this country also worsened during this period, from 3.09 gha in 1994 to 4.74 gha in 2007, showing higher pressure on the nature. Borucke et al. [51] reported the EFA calculation of all countries of the world in detail with the ranks of each country in comparison with others. The main result of the study attributed to ecological deficit or ecological reserve of each country which could be concluded from the ratio of BC to EF. It is important to judge from the sustainability status of each nation by the ration of BC/EF of that country.

### 1.2. Theoretical framework

**Fig 1:**
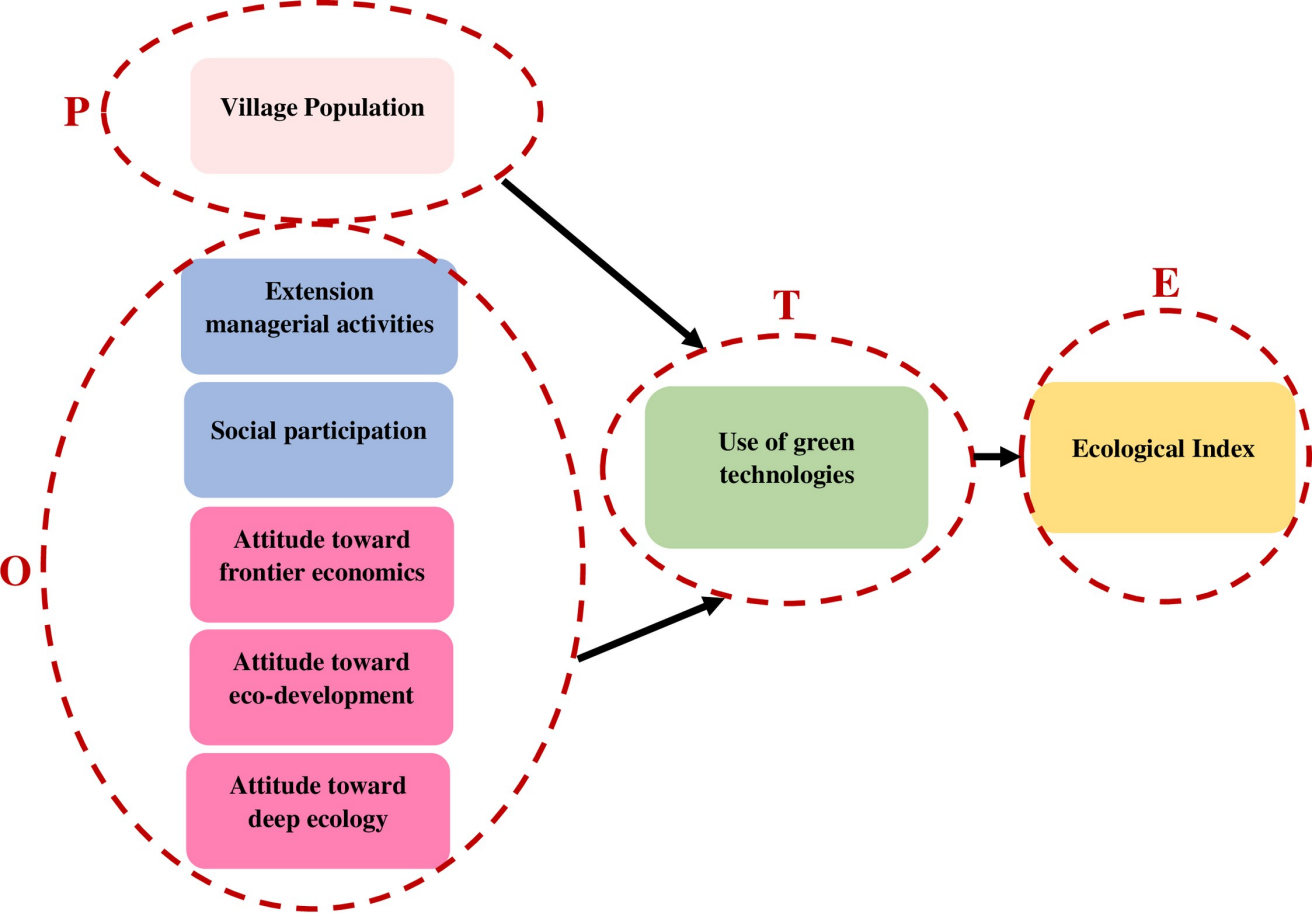
Theoretical model of the study adopted from POET model.

Since humankind has a key role on the environment pressure in terms of natural resources consumption in different settings such as agricultural and livestock activities, the calculation of ecological indices including BC and EF of rural areas was the main purpose of this study. First, the ecological indicator and its components of the studied villages were computed, then the rural areas were categorized and compared based on their sustainability scores and compared. Next, a causal model of factors affecting the ecological index was presented. The conceptual model of this phase was mainly designed by the human ecology model of Duncan in 1961 called POET which enables one to see the complex, reciprocal relationships among population, organization, environment as well as technology. As it is seen in the research theoretical model (Fig 1), “*villages population*” is considered as independent variable due to POET model, five other variables including “*extension managerial activities*” and “*social participation*” [52, 53] which shows the extension experts relationships with their target groups (farmers and rural people) and also the attitudes of rural households toward different environmental management paradigms of “*frontier economics*”, “*deep ecology*” and “*eco-development*” [8, 54] are represent as the extension organization considered as independent variables in the study model. Moreover, “*use of green technologies*” was another factor of POET model of technology. It was considered as the mediator variable of the use of green technology which linked the research variables to the ecological indicator. According to Ecological Modernization (EM), using eco-friendly techniques like precision technologies in agriculture sector is one of the best solutions for environment and natural resources conservation in sustainable agricultural development [55]. Finally, environment is the last components of Duncan model which has been considered as ecological index (dependent variable) in the model. Overall, the theoretical model of current study was presented in Fig 1, indicating factors influencing ecological index of villages adopted from POET model.

## 2. Research method

Achievement of the main goal of the study, i.e., calculation of ecological indices at the rural level, as well as comparing rural society in term of social-ecological conditions, factors affecting the ecological index and determining the paradigmatic perspective of the managers and policy makers of agricultural development of Fars province due to environmental management, required using a combination of methods, techniques and statistical software in two principle steps.

### 2.1. First step- calculation of ecological indices at rural level

**Fig 2:**
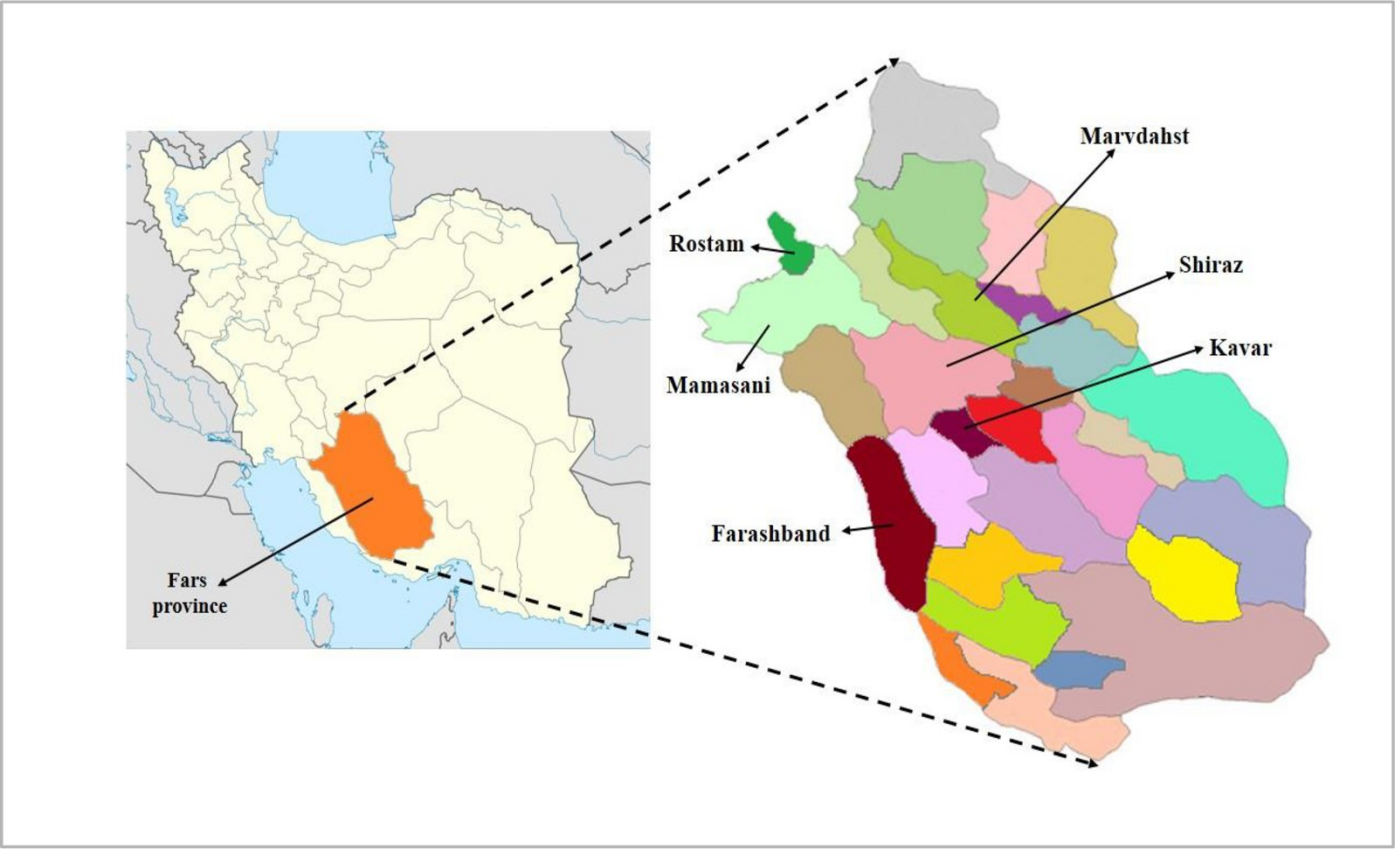
Geographical position of the research areas.

This research phase was conducted in the rural areas of Fars province using both survey and interview for data collection. Multi-stage stratified random sampling method was used to determine the study sample. Six counties of Fars province, including Shiraz, Marvdasht, Kavar, Mamasani, Rostam and Farashband, were selected randomly (Fig 2). Since the unit of study was the village, a list containing the names of all of the villages of those six counties was provided in addition to the population and rural household number of each village. Consistent with the importance of a population factor on society’s consumption of environment and natural resources, villages with a population of less than 50 households were omitted. Then, 50 villages of the mentioned counties were selected randomly in accordance with the special sampling formula (Eq 1) [56] in order to determine accounting of ecological indices at the rural level.

$$\begin{array}{l} \;n=N\;\delta^{2}/\left(N‐1\right)D+\delta^{2}\\ n=(602)(50.649)/(601)(1)+(50.649)=49.79≅50\\ B=D^{2}/4=(2{)}^{2}/4=1 \end{array}$$ (1)

where:

*N* = Total numbers of the villages in research area

*n* = Sample size

δ^2^ = Variance of the sample (using the results of the pilot study)

*B* = Estimated error which is assumed 2 in this study.

Two separate questionnaires were designed in order to calculate ecological indices, including BC and EF. The first one, as a rural questionnaire, includes the required data of the whole village for assessment of ecological indices. The required data for the studied villages within the 12 Agricultural Extension and Service Centers (AESC) of Fars province were gathered. Some of the villages are covered and controlled by each AESC and every county contains some AESC in different rural areas (The main formal agricultural system in Iran is centralized system dependent to government which means there is a Ministry of Agriculture Jihad in the capital of the country and then there are some Organizations of Agriculture at provincial level, some agricultural offices at county level of each province and finally there are some agricultural extension service (AES) centers at the lowest level that has direct communication with rural areas and people. So, all of the key agricultural decisions, policies and programs are made at higher level and then trickle down for implementation. It is important for the research team to calculate and compare the ecological indices (EF/BC) at different top-down levels of national, counties, centers (called Dehestan) and village. EF/BC at national levels are calculated annually by an international institution called Global Footprint Network (GFN), and their report is published worldwide, therefore the study focused on calculating these index at rural level, then one higher systematic level at agricultural centers *(some special villages are covered by an agricultural center)* and then another higher level at counties *(some agricultural centers are monitored by an agricultural office at county level)*. Indeed, different scales of village, center and county had been considered to discover even small differences in sustainability score. For example, we could see two sustainable villages at rural scale calculation. But it was decreased to only one sustainable service center and there was not any sustainable county at larger level. Thus, we would neglect the small differences if we do not consider village or center levels.). Complementary information was verified and completed by referring to different organizations including the Organization of Agriculture Jihad of Fars province, Central Office of Natural Resources and Watershed of the province as well as the Fars Regional Water Authority. The second questionnaire, i.e., the farmers’ questionnaire, contains the required data in terms of rural household consumption of agricultural inputs and products, natural resources as well as fossil fuels to complete the necessary data for ecological indicator accounting. The questionnaire also includes the data for assessing other variables such as social participation and communication of rural society with agricultural extension organizations, farmers`attitudes toward different environmental management paradigms in agriculture, accessibility and application of agricultural green technologies and the demographic characteristics of respondents. Completing the second questionnaire, based on the population of each village, a random sample of 423 farmers of 50 villages were selected due to the special sampling formula and the data were collected by interviewing farmers. The study was reviewed and approved by the Higher Education Committee of the School of Agriculture as well as the Higher Education Committee of Shiraz University before the study began.

Total BC is determined by calculation of five land use types including cropland, grazing land, marine area, forest and infrastructure area. For a given country, city or a village, the BC is calculated as follows [51]:

$$BC_{}=\sum A_{N,i}\times YF_{N,i}\times EQF_{i}$$ (2)

Where A_N,i_ is the bioproductive area that is available for the production of each product i (*ha*) at the country, city or village level, YF_N,i_ is the country-specific yield factor for the land producing products i (*t/ha*), and EQF_i_ is the equivalence factor for the land use type producing each product i [51]. The same formula is used for each land use type and then aggregated for determining total BC.

The EF comprises production from the six types of cropland, grazing land, marine area, forest, carbon uptake land and infrastructure area that the two groups of forest product and carbon sequestration compete for the same BC category of forest land. The main formula for EF assessment is described as follows (All six types of land use in total EF and total BC was calculated as it is defined in the formula 2 and 3. It should be mentioned that the studied area (Fars province) does not have any area attributed to Marine type, thus this land use type equaled zero for this province. It is almost the same for the Forest type as well. So, the rest land use types including Agriculture (cropland), Pastures (grazing-land), Urban areas as well as Carbon footprint were calculated. Total BC and total EF of all studied region was calculated and reported during the paper through the text, tables and graphs, and due to the main focus of the research on rural households and farmers, only the two main related types of agriculture and pasture were reported in detail and separately.). For a given nation, city or village the EF of production, EF_P_, represents primary demand for BC and is calculated as [51]:

$$EF_{P}=\sum \left(P_{i}/Y_{N,i}\right)\times YF_{N,i}\times EQF_{i}=\sum \left(P_{i}/Y_{w,i}\right)\times EQF_{i}$$ (3)

Where P is the amount of each primary product i that is harvested *(tone)* (or carbon dioxide emitted) in the nation, city or village; Y_N,i_ is the annual national average yield for the production of commodity i *(t/ha)* (or its carbon uptake capacity in cases where P is CO_2_); YF_N,i_ is the country specific yield factor for the production of each product i (tone per hectare); Y_w,i_ is the average world yield for commodity i *(t/ha)*; and EQF_i_ is the equivalence factor for the land use type producing products i [57, 58]. The same equation is used for each category of cropland area, grazing land area, marine area, forest area and infrastructure area. The aggregate of these equations equals total EF.

The validity of the questionnaires has been verified by a panel of experts and specialists from two departments of Shiraz University including Agricultural Extension and Education and Environment and Natural Resources as well as the research team of Global Footprint Network in the U.S.A. The reliability of the questionnaires was verified by a pilot study with 40 farmers out of the main sample of the study from the rural areas of Sepidan County. Cronbach`s alphas of research variables were computed as follows: Social participation (0.74), use of green technologies (0.72), extension managerial activities (0.75), attitude toward paradigmatic perspectives of frontier economics (0.84), eco-development (0.87) and deep ecology (0.77). The mathematical calculations for the BC and EF formulas with collected data were performed using Excel and SPSS in order to develop ecological indices accounting. A separate Excel file was made for each village. Some other statistical analyses including analysis of variance (ANOVA) were done by SPSS_21_ in order to compare different studied villages categorized by BC/EF ratio index. Finally, a Structural Equation Model (SEM) and path analysis was drawn to determine factors affecting the ecological indicator using AMOS_20_.

### 2.2. Second step- AHP of policy makers’ perspective

The AHP technique was used to determine the paradigmatic perspective of agricultural policy makers and agricultural extension managers of Fars province in terms of environmental management. The research sample of this phase of the study comprises two groups of top managers of agricultural extension as well as the policy makers and authorities of the Organization of Agriculture of Jihad of Fars province.

The organization of Agriculture Jihad of Fars province controls the agricultural executive activities at county, district and rural levels of the province; they act as the highest organization of the Ministry of Agriculture Jihad and other executives sector at the provincial level. This organization is located in the center of Fars province, Shiraz. One of the groups of this phase of the study includes Agricultural managers and experts of the Organization of Agriculture Jihad (AOAJ). The other group includes managers and authorities of Agricultural Extension of Organization of Agriculture Jihad (AEOAJ). At the provincial level, the agricultural extension tasks are done by the ECM which follows-up the policies and main programs from the ministry. This sector also has the task of coordination among specialized sectors and operational units at county and rural levels, especially the agricultural extension and service centers. A total of 22 managers who are the key authorities of these two groups of the organization were interviewed.

The questionnaire of this phase of the study was designed in three steps as follows. First, based on review of scientific sources and related literature of environmental management paradigms, 63 different components were listed as indicators of environmental management. Next, similar components were merged and new appropriate concepts were incorporated based on the research team’s assessments. Finally, 35 components in 4 main groups of economic, social-cultural, environmental and technological-political areas were categorized after incorporating and summarizing the first list. To gather the viewpoints of different groups of experts, scientists and scholars of environmental management in agriculture, a total 62 questionnaires were completed by different groups including managers and specialists of the Central Office of Environmental Protection of Fars province and elites of the think tank of this Office, professors of the School of Agriculture of Shiraz University (Departments of Environment and Natural Resources and Agricultural Extension and Education) as well as the Global Footprint Network in the U.S.A., the members of the Council of Engineering System Organization of Agriculture and Natural Resources of Fars province and the managers and executives of the Organization of Agriculture Jihad of Fars province. In the primary questionnaire, the respondents were asked to rank and weight the components of each category in the spectrum of 1 to 5 based on the importance of each component in sustainable environmental management of agriculture. Based on Q-methodology [59, 60], they also were instructed not to assign the same weight to the majority of the components, so that the same weight could not be assigned to more than two components of every category. The scientists were asked to merge similar components or add new ones which did not exist in the questionnaire.

Finally, the hierarchical model of AHP was developed. Determination of an appropriate paradigm of environmental management for sustainable agricultural development was the principal goal of the model. Based on the findings of the primary questionnaire, the 9 ultimate criteria of environmental management (human basic needs, economic dependency on natural resources, environmental ethics and culture, rational use of resources, equity and poverty alleviation, eco-friendly technologies, biodiversity, environmental adaptability, mutual collaboration and participation) were selected as the key components and were placed in the second row of the tree model. The final questionnaire of AHP using 9 criteria was designed in the format of a pairwise matrix to compare two by two regarding the priority of each criterion in the spectrum of 1 to 9. Frontier economics, deep ecology and eco-development are the three paradigmatic perspectives that comprised the third branch of the hierarchical model. The collected data from the second step were analyzed by Expert choice_11_.

## 3. Results and discussion

### 3.1. Calculation of ecological indices

#### 3.1.1. Rural level

The results of the calculations of BC and EF of 50 studied villages are shown in Table 1, since the accounting of these two parameters and the comparison of different villages in terms of BC and EF was the main purpose of the study with the stated goal to determine the sustainability situation of the studied regions. The ratio of BC to EF has been calculated as well. This ratio shows the pressure that has been imposed on the environment by natural resources consumption of the people who live in each village. If the ratio is higher than one, it means that the village has ecological reserve and the consumption of inhabitants is less than the BC of the village. A village with ecological reserve could be categorized as a sustainable area in terms of natural resources consumptions. On the other hand, if the ratio of BC to EF is less than one, it indicates natural resources exploitation which means the pressure on the environment has exceeded the BC and ecological deficit would result. These villages could be categorized as an unsustainable condition. Finally, when the BC to EF ratio is equal to one, it means that the village is in a balanced situation in which consumption of natural resources (EF) equals BC. The third group could be also categorized as a relatively sustainable condition.

**Table 1:** Results of ecological indicators including BC, EF, BC/EF and the ranks of studied villages in Fars province.

Rank	Village name	BC	EF	BC/EF	Rank	Village name	BC	EF	BC/EF
1	Norouz Abad	16.89	10.30	1.64	26	Maghsoud Abad	0.79	2.40	0.33
2	Chenar-mishowan	3.17	2.52	1.26	27	Hossein Abad	0.77	2.63	0.29
3	Dejgah	2.28	2.32	0.98	28	Ezz Abad	1.07	4.00	0.27
4	Mosghan	1.67	1.74	0.96	29	Shahrak Valiasr	1.00	3.69	0.27
5	Romaghan	2.00	2.31	0.87	30	Mouraki	0.32	1.34	0.24
6	Tarbor Jafari	1.26	1.48	0.85	31	Genjan	1.08	4.62	0.23
7	Gouri	2.23	2.62	0.85	32	Shams-abad Borzoo	0.83	3.62	0.23
8	Doudej	1.29	1.53	0.84	33	Kouh Sabz	0.27	1.17	0.23
9	Dehno	2.25	2.74	0.82	34	Kedenj	1.96	8.74	0.22
10	Johari	2.11	2.87	0.74	35	Bajgah	0.52	2.37	0.22
11	Band-e-amir	3.07	4.39	0.70	36	Koushk	0.41	2.01	0.20
12	Bagdaneh	2.02	3.07	0.66	37	Mozaffari	0.58	2.95	0.20
13	Izad-khast Baseri	0.85	1.31	0.65	38	Kenareh	0.41	2.35	0.17
14	Mansour Abad	1.37	2.38	0.58	39	Tal-rizi Alivand	0.56	3.46	0.16
15	Ahmad Abad	1.51	2.77	0.55	40	Haji Abad	0.73	4.47	0.16
16	Khoushab	6.68	12.28	0.54	41	Dashtak	0.32	2.04	0.16
17	Dehno Sadat	1.77	3.47	0.51	42	Tasouj	0.36	2.48	0.15
18	Shoul	1.02	2.01	0.51	43	Zangi Abad	0.31	2.27	0.14
19	Badaki	1.01	2.25	0.45	44	Fahlian	0.52	3.72	0.14
20	Dolat abad	1.31	3.03	0.43	45	Gachgaran	0.35	2.64	0.13
21	Moraskhan Olia	1.76	4.40	0.40	46	Norouzan	0.57	4.50	0.13
22	Kenar Malek	0.70	1.85	0.38	47	Foroud	0.63	5.12	0.12
23	Kouraki	1.42	3.89	0.37	48	Shams-abad Takht	0.35	2.90	0.12
24	Rahmat Abad	0.93	2.71	0.34	49	Fath Abad	0.28	2.65	0.11
25	Mehrian	2.33	7.09	0.33	50	Garm Abad	0.46	4.94	0.09

• The unit of all of the indices: global hectare (gha).

According to the results of Table 1, 96 percent of the villages have been graded as meeting unsustainability criteria, while the ecological indicator (BC to EF ratio) of only two of them is greater than one. The ecological index of 16 villages (32 percent) was calculated as between 0.5 and 1 and it was less than 0.5 for the others. Thus, the condition of studied villages has been in the range of unsustainable to quite unsustainable, since most of them have been in a critical condition environmentally and the pressures which have been imposed on the natural resources by the inhabitants are greater than the ecological capacity of those regions. The villages have been ranked due to the ecological index as well (Table 1).

**Fig 3:**
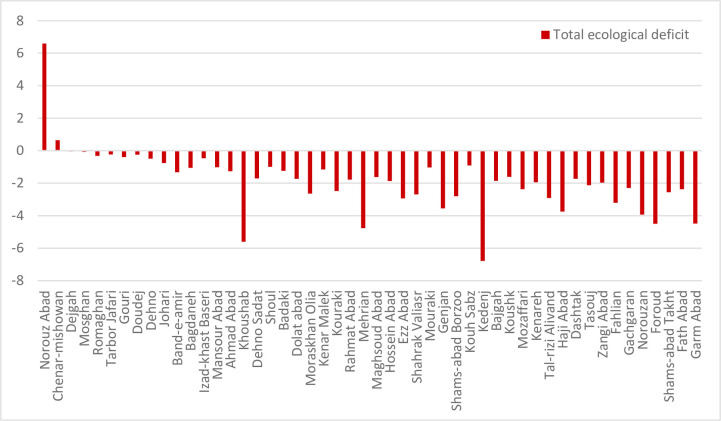
Total ecological deficit (BC-EF) of the villages. Ecological deficit refers to the amount of EF that higher than BC of the region.

The difference between BC and EF represents the pressure or exploitation of humans on the BC of each region. Ecological reserve would pertain if the difference of BC and EF were a positive number and the negative amount indicates the ecological deficit. The differences between the ecological indices of 50 studied villages is shown in Fig 3. There are only two villages located above the horizontal axis and the others are sorted in the range of unsustainability under the horizontal axis. Revieweing the rurals`situation indicated that the two villages with bettter sustainability score has lower access to the city and the center of the province (Shiraz), these villagers live in more traditional condition with lower consumption. In other hand the rurals with worse sustainability situation in the list have more commuting to the cities with semi-urban lifestyle. Some industrial sites exist near the village and different land use changes had been seen in these areas due to their better climates as attracting urban dwellers to move there as hobby farming in their leisure time, so these parameters could have effect on their sustainability score. This simillar findings was confirmed in Fatemi et al. [61] for this province as well.

As noted in the research method section, BC and EF include six different types of human natural resources consumption. The results of two categories of agriculture and pasture have been reported in accordance with the emphasis of the study (Table 2). Accounting the ratio of BC to EF in the agricultural sector indicated that there was only one village (Dodej) with a ratio of more than one and this amount was equal to a ratio in another village (Mosghan). This ratio was less than one in the remaining villages; this means that the pressure of the inhabitants on the environment and natural resources in terms of agricultural activities was greater than their BC. Reviewing the results of the pasture sector showed a different situation. Eighteen cases (36 percent) of villages were categorized in a sustainable situation (with environmental ratio of more than one), and the others were in the range of unsustainable (less than one). Therefore, the agricultural activities could be categorized as in a critical situation in comparison with the pasture and livestock sector.

**Table 2:** Results of BC/EF ratio index of cropland and grazing land of villages in Fars province.

No	Village name	BC/EF of Cropland	BC/EF of Grazing land	No	Village name	BC/EF of Cropland	BC/EF of Grazing land
1	Dodej	1.11	0.46	26	Dejgah	0.38	14.00
2	Mosghan	1.00	1.22	27	Maghsoud Abad	0.37	0.08
3	Romaghan	0.91	6.26	28	Kenareh	0.35	0.01
4	Izad-khast Baseri	0.86	0.42	29	Gouri	0.35	15.80
5	Ezz Abad	0.85	0.04	30	Shahrak Valiasr	0.34	0.01
6	Bajgah	0.65	0.15	31	Mehrian	0.33	0.01
7	Badaki	0.65	1.83	32	Rahmat Abad	0.33	0.04
8	Khoushab	0.65	0.20	33	Mouraki	0.33	0.14
9	Chenar-mishowan	0.63	7.59	34	Zangi Abad	0.29	0.08
10	Hossein Abad	0.62	0.09	35	Garm Abad	0.29	0.01
11	Tarbor Jafari	0.61	1.93	36	Shams-abad Borzoo	0.28	0.01
12	Kedenj	0.56	0.70	37	Dehno Sadat	0.27	4.64
13	Band-e-amir	0.55	1.12	38	Koushk	0.24	0.03
14	Norouz Abad	0.53	7.72	39	Ahmad Abad	0.23	5.38
15	Kenar Malek	0.52	0.19	40	Mansour Abad	0.22	5.89
16	Bagdaneh	0.51	0.64	41	Moraskhan Olia	0.22	4.00
17	Johari	0.47	4.14	42	Gachgaran	0.21	0.17
18	Kouraki	0.46	0.03	43	Mozaffari	0.19	0.18
19	Dolat Abad	0.45	2.20	44	Tasouj	0.18	0.09
20	Fahlian	0.44	0.11	45	Fath Abad	0.18	0.01
21	Shoul	0.42	1.44	46	Tal-rizi Alivand	0.17	0.13
22	Kouh Sabz	0.41	0.33	47	Haji Abad	0.16	0.20
23	Genjan	0.39	6.88	48	Dashtak	0.13	0.21
24	Dehno	0.39	12.14	49	Norouzan	0.12	0.15
25	Shams-abad Takht	0.38	0.03	50	Foroud	0.11	0.36

• The unit of all of the indices: global hectare (gha).

**Fig 4:**
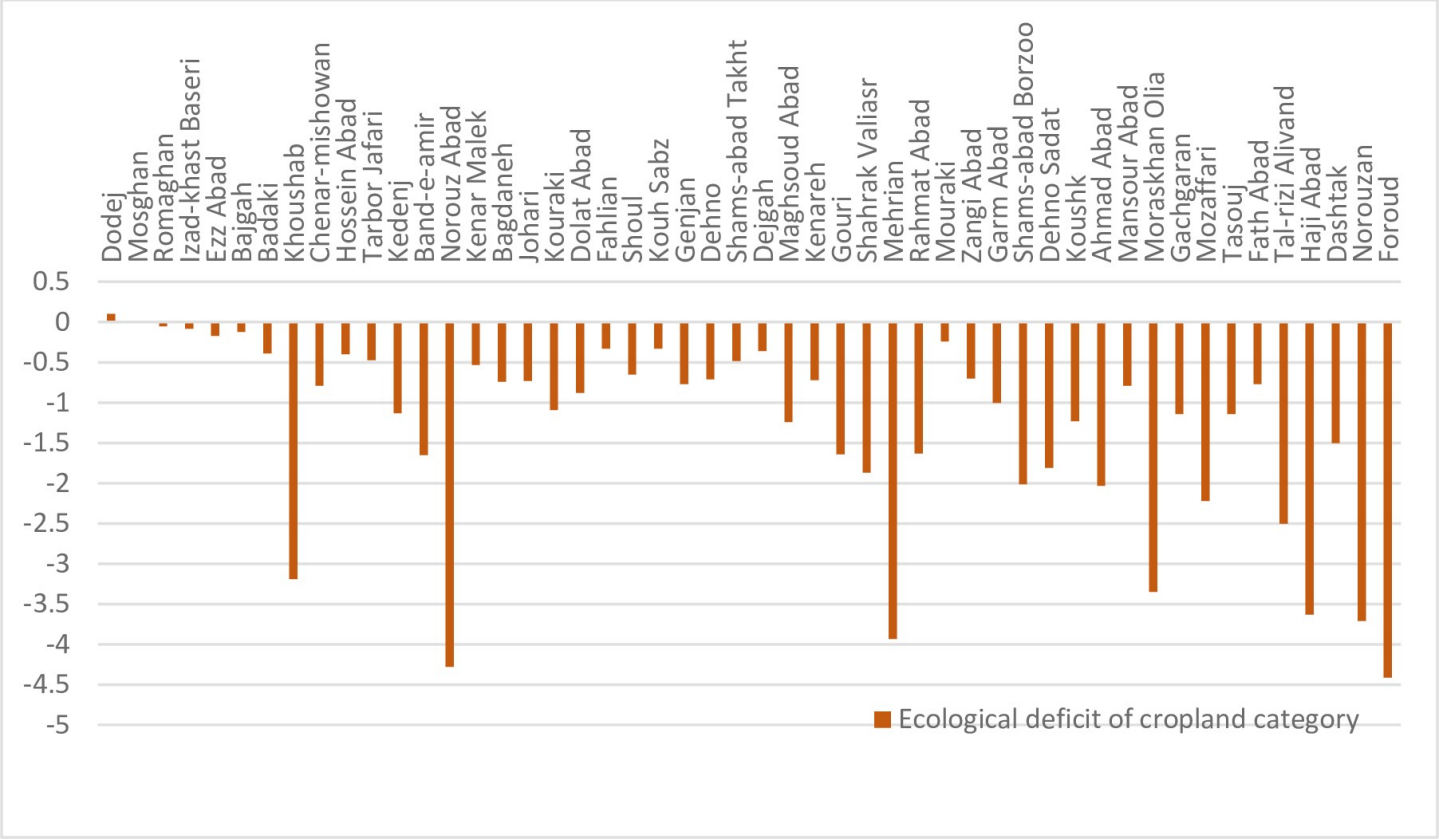
Ecological deficit (BC-EF) of cropland category of the villages. Ecological deficit refers to the amount of EF that higher than BC of the region.

**Fig 5:**
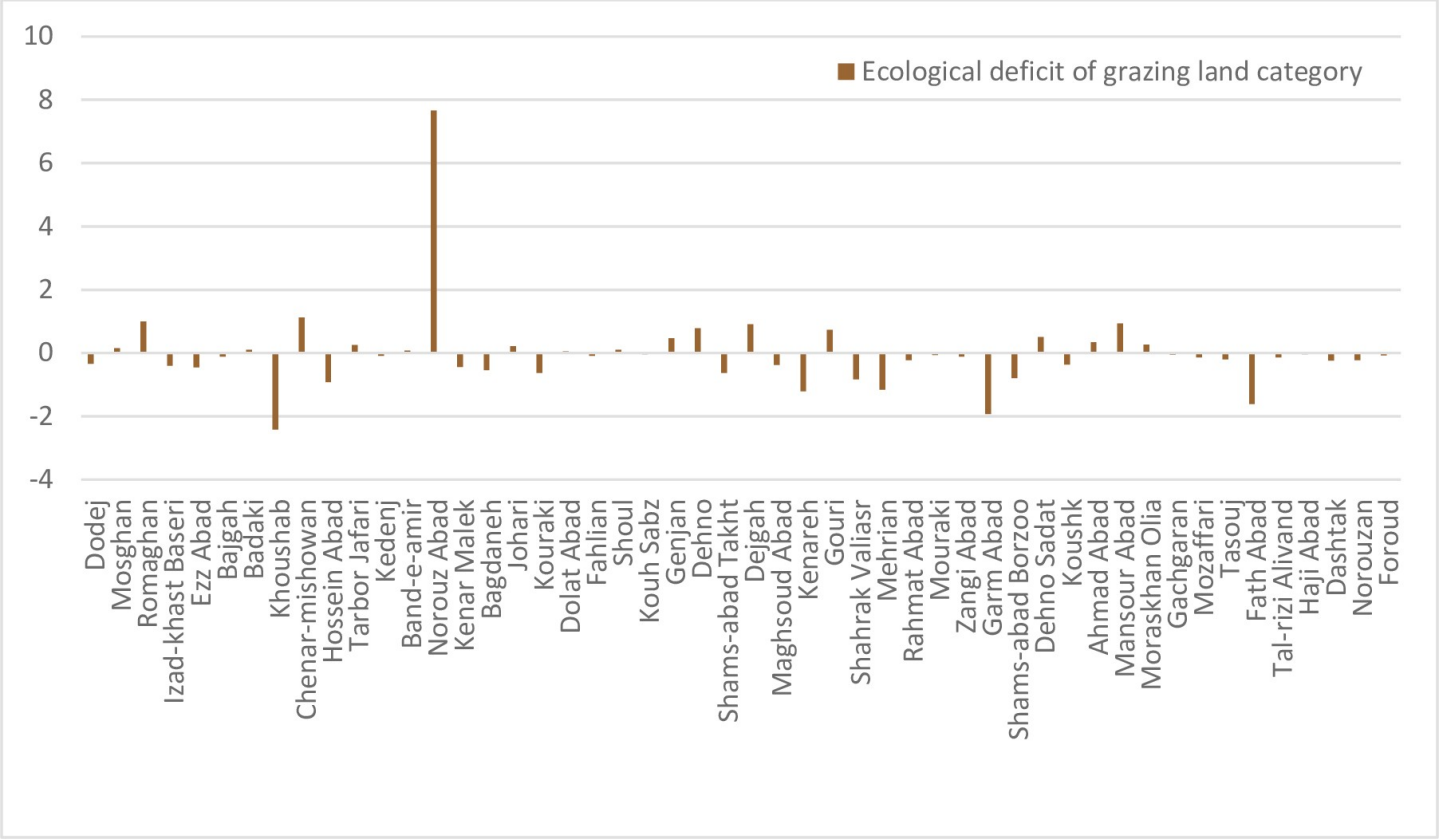
Ecological deficit (BC-EF) of grazing land category of the villages. Ecological deficit refers to the amount of EF that higher than BC of the region.

The ecological deficit of agriculture and pasture (BC and EF difference) are shown in Figs 3 and 4, respectively. Comparison of these Figs clearly indicates the unsustainable condition of the villages in terms of the pressure of agricultural activities on natural resources. Most villages are located under the horizontal axis in the negative range. Fig 5 shows that most villages are scattered around the horizontal axis and 18 cases (36 percent) are above zero when evaluating the pasture sector. The results of agriculture sector showed different condition in the studied rural areas. Indeed, most of them had inappropriate situation as natural resources overconsumption in the cropland type. It is due to the dominant economic activities of villages in Fars province and Iran. Actually, village in Iran is defined as an area which agriculture is the only or the main job of their habitants. This is true for most of studied rural, so the eco-deficit is anticipated in the cropland due to the high pressure on the environment and different natural resources (especially lands and water) by the people through their cultivations.

#### 3.1.2. AES centers level

**Fig 6:**
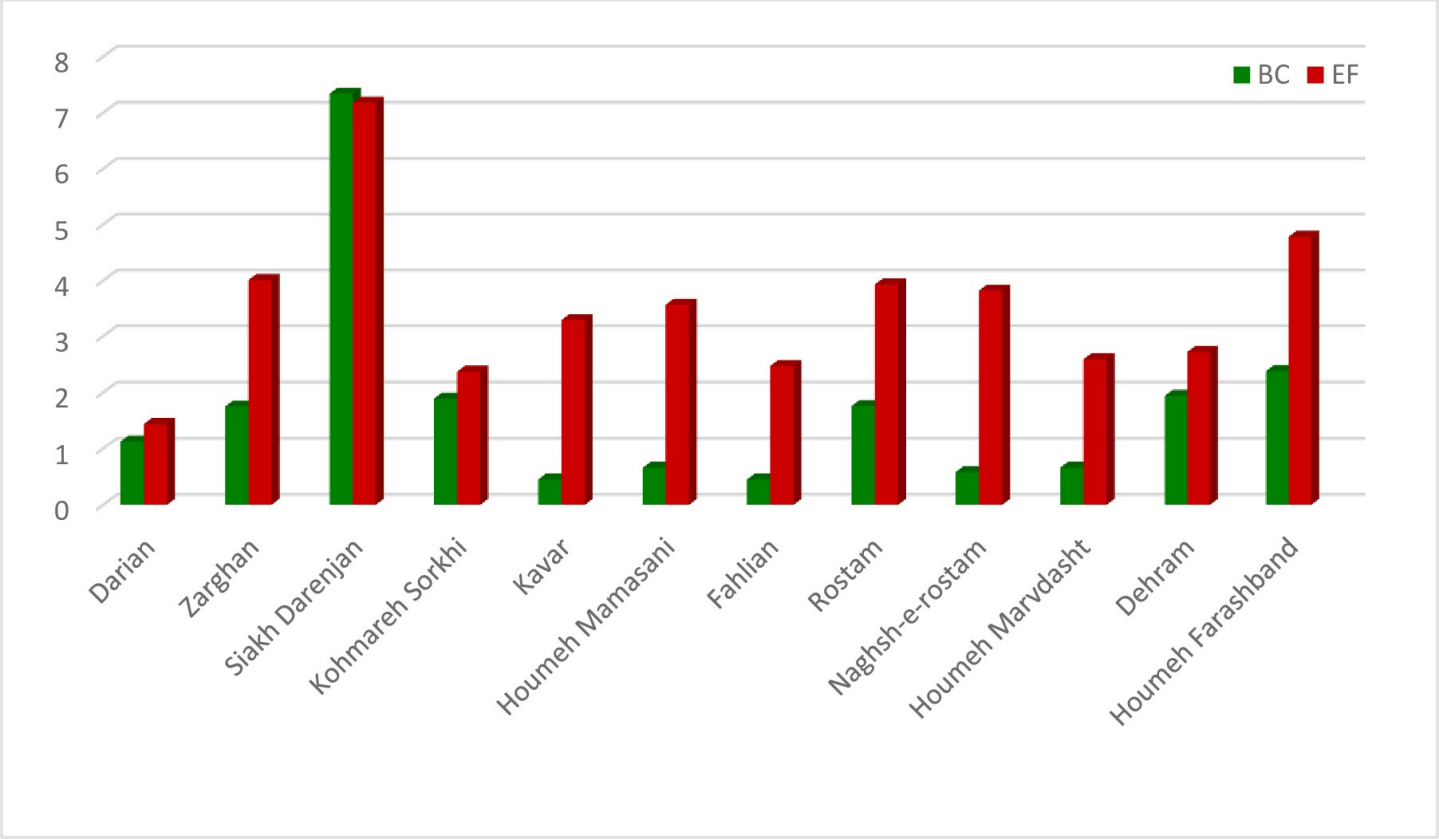
Total BC and EF of the service centers in Fars province.

Detailed comparison of the sustainability status of the villages is presented followed by discussion of the situation of the areas covered by each agricultural extension and service center. These centers are the executive organization of agricultural extension at the rural level and are the direct liaison of the Ministry of Jihad Agriculture and the farmers at the local level. All of the programs which are designed by the agricultural specialists at the ministry level are implemented by the experts of AESC [62]. The total sample of villages (50 villages) are covered by 12 agricultural extension and service centers. The findings of BC and EF accounting in 12 service centers are shown in Fig 6. All of the service centers except Siakh Darenjan had ecological deficit indicating that the consumption of the inhabitants (EF) was greater than the BC.

The results of ecological indices calculations including the ratio of BC to EF as well as the difference between BC and EF of every extension and service center are presented in Table 3. Findings in all types of land use revealed that all of the service centers except Siakh Darenjan were in an unsustainable condition, as the BC/EF ratio of these centers were less than one and the difference of the two indices was negative. This ratio equaled 1.04 for the service center of Siakh Darenjan which is located at the break-even point in terms of natural resources consumption. If this trend continues by increasing pressure on resources, the condition of this service center would be similar to the others in near future. The rural areas of Siakh Darenjan are located in the hillside, it is not easy to commute to Shiraz for the most of villages, they have more traditional lives than the other studied rural areas. The two villages with ecological reserve in the previous section were the subset of Siakh Darenjan`s service center. Overall, this area has an appropriate sustainability condition in terms of total BC/EF ratio due to the reasons which has been described as the characteristics of their environment and people, and it is mentioned in the previous section as well. But when we focus on agriculture sector, Siakh Darenjan had also ecological deficit in cropland which indicated the farmers had high pressure on the lands, water and other types of resources through their farming activities as their main economic task.

**Table 3:** The BC/EF ratio index and ecological deficit of the service centers in Fars province.

No	Service center	Total		Cropland		Grazing land	
		BC/EF	Eco-deficit	BC/EF	Eco-deficit	BC/EF	Eco-deficit
1	Siakh Darenjan	1.04	0.16	0.55	-2.07	6.47	2.9
2	Komareh Sorkhi	0.80	-0.48	0.70	-0.27	1.27	0.21
3	Darian	0.78	-0.31	0.83	-0.15	0.68	-0.17
4	Dehram	0.72	-0.78	0.36	-1.05	9.5	0.51
5	Houmeh Farashband	0.49	-2.4	0.60	-1.23	0.40	-0.71
6	Rostam	0.45	-2.17	0.24	-2.58	4.54	0.39
7	Zarghan	0.44	-2.26	0.41	-1.79	0.59	-0.16
8	Houmeh Marvdasht	0.25	-1.93	0.42	-0.80	0.07	-0.47
9	Fahlian	0.19	-2.02	0.47	-0.23	0.11	-0.08
10	Houmeh Mamasani	0.17	-2.91	0.23	-1.47	2.11	0.1
11	Naghsh-e-rostam	0.15	-3.24	0.24	-1.84	0.01	-0.71
12	Kavar	0.14	-2.84	0.14	-2.34	0.06	-0.41

• The unit of all of the indices: global hectare (gha).

**Fig 7:**
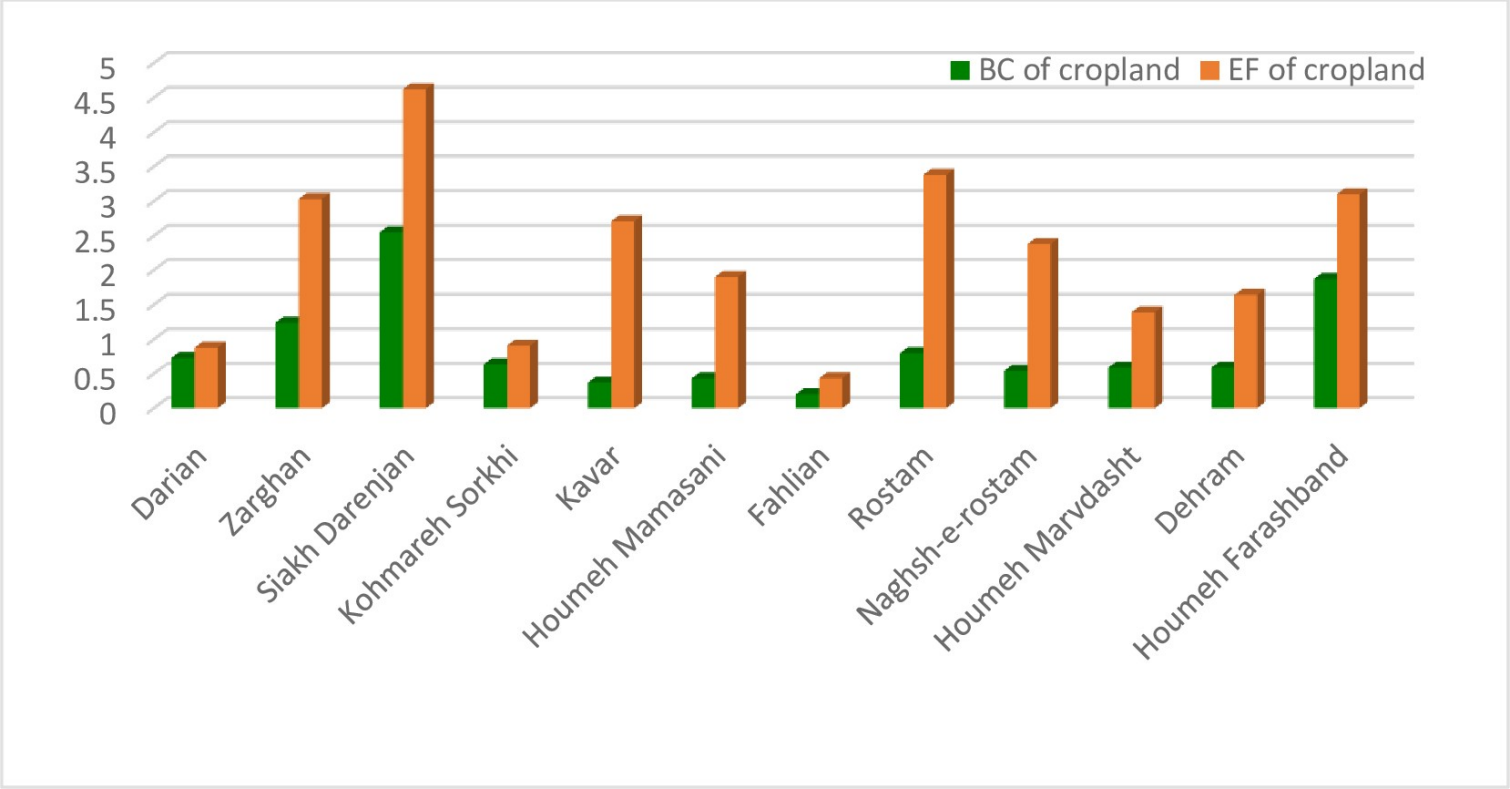
BC and EF of cropland of the service centers in Fars province.

The presentation of the total ecological indices of service centers is focused on the calculation of BC and EF of agriculture and pasture. The BC and EF of agriculture in different service centers are shown in Fig 7. The farmers`consumption of natural resources in all service centers exceeds their ecological capability as the EF of all 12 centers is greater than the BC. This indicates society’s pressure on the environment and natural resources via agricultural activities. Indeed, the harvesting of agricultural products by the farmers of these areas was higher than the region’s capacity in terms of agriculture. The ratio and difference of the two ecological indices in different extension and service centers are presented in Table 3. All of the agricultural extension and service centers had ecological deficit with the BC/EF ratio of less than one based on the results of indices accountings. The BC/EF ratio of four of the service centers (34 percent) were between 0.5 and 0.8 and this ratio was less than 0.5 for the other eight centers (66 centers). The difference of indices was also negative for all of the extension and service centers in the agricultural sector.

**Fig 8:**
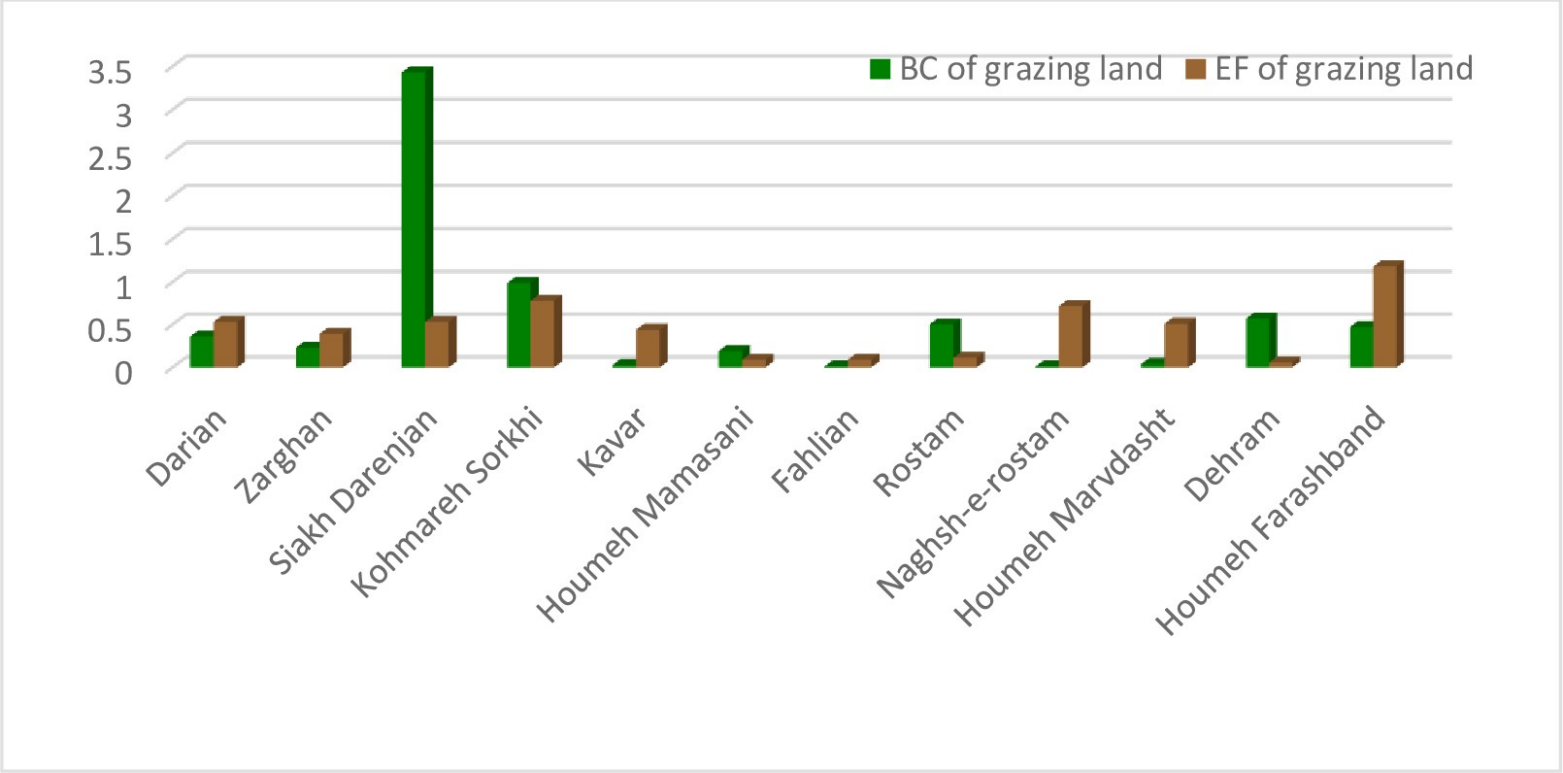
BC and EF of grazing land of the service centers in Fars province.

The results of EF and BC accounting of pasture for different extension and service centers are shown in Fig 8. The other findings including BC/EF ratio and the difference of these two indices regarding pasture are presented in Table 3. Based on the results, the pasture condition of 5 service centers (42 percent) were sustainable and the other 7 centers (58 percent) were categorized in the unsustainable range with a negative ecological index less than one. The service centers of Dehram, Siakh Darejnjan, Rostam, Houmeh Mamasani and Kohmareh Sorkhi had ecological reserve in terms of pasture sector while the other centers had an ecological deficit.

#### 3.1.3. County level

**Fig 9:**
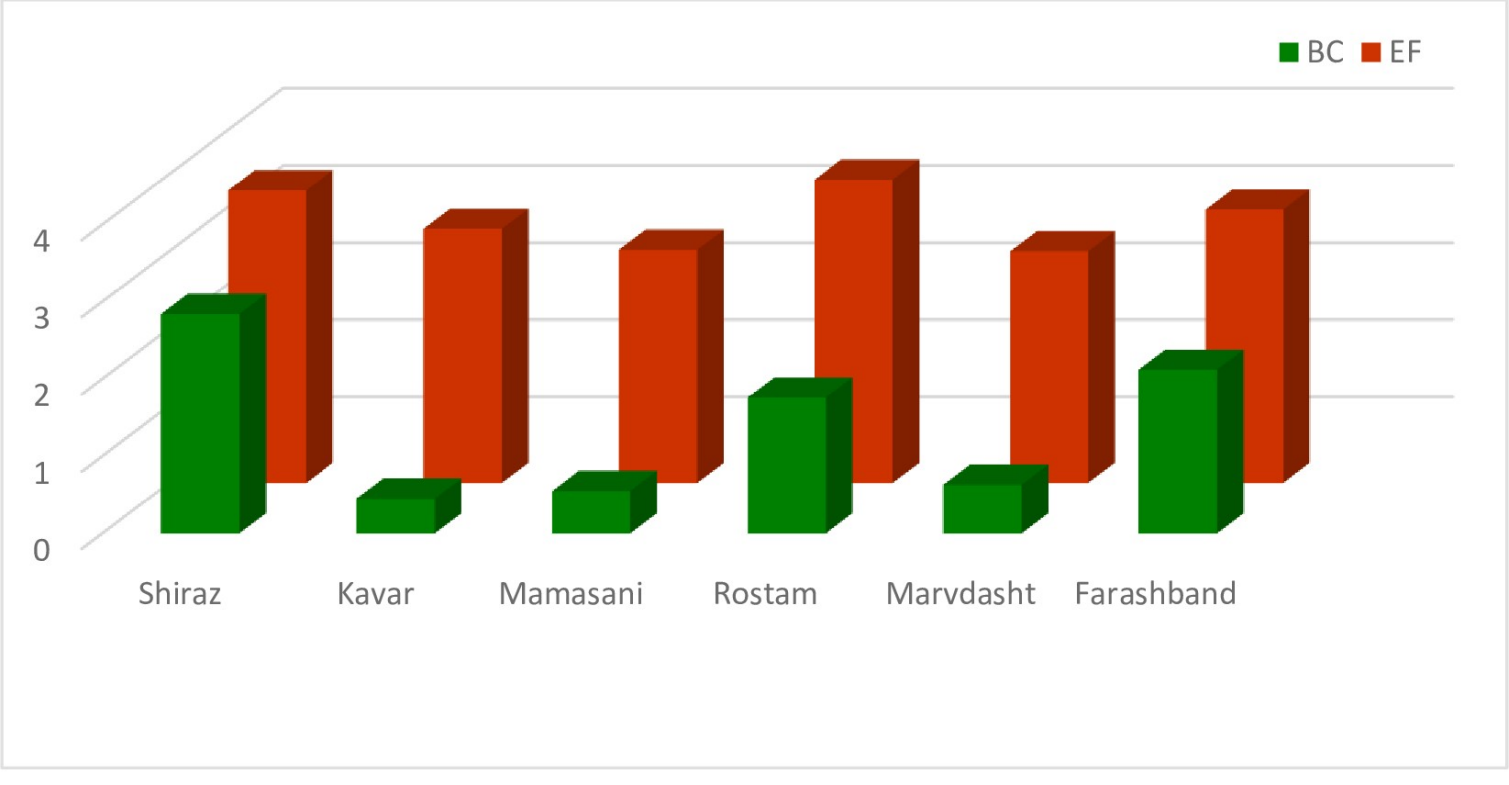
Total BC and EF of the counties in Fars province.

The ecological indices of Fars province were calculated at the county level. The results of total BC and EF of all of the six counties are shown in Fig 9. The EF of all counties was greater than their BCs indicating an unsustainable condition. Indeed, the consumption of natural resources of these counties was greater than the regenerative rate of resources resulting from high pressure on the environment and natural resources by different societal activities.

The ratio and difference of BC and EF are presented in Table 4. All of the counties had ecological deficit as evidenced by the negative value of the differences between these two indices. As for the ecological index, the BC/EF ratio was also less than one for all of the counties; two cases were categorized in the range of unsustainable with ratios between 0.5 and 0.7 and the ratio was less than 0.5 for the other four counties. The results indicated an unsustainable condition of the environment for the counties of Fars province, suggesting that providing applicable and executive programs and policies is required in order to change the current trend. Indeed, as we expand the scale of analysis from the village to the county, the differences could not be seen. We had two sustainable villages and one sustainable service center but we do not have any sustainable county based on the results. It is the same condition for the cropland type of all the counties as well.

**Table 4:** The BC/EF ratio index and ecological deficit of the counties in Fars province.

No	County	Total		Cropland		Grazing land	
		BC/EF	Eco-deficit	BC/EF	Eco-deficit	BC/EF	Eco-deficit
1	Shiraz	0.75	-0.95	0.52	-1.17	1.82	0.5
2	Farashband	0.60	-1.42	0.49	-1.12	1.04	0.02
3	Rostam	0.45	-2.17	0.24	-2.58	4.54	0.39
4	Marvdasht	0.21	-2.37	0.33	-1.15	0.05	-0.55
5	Mamasani	0.18	-2.47	0.27	-0.85	1.11	0.01
6	Kavar	0.14	-2.84	0.14	-2.34	0.07	-0.41

• The unit of all of the indices: global hectare (gha).

**Fig 10:**
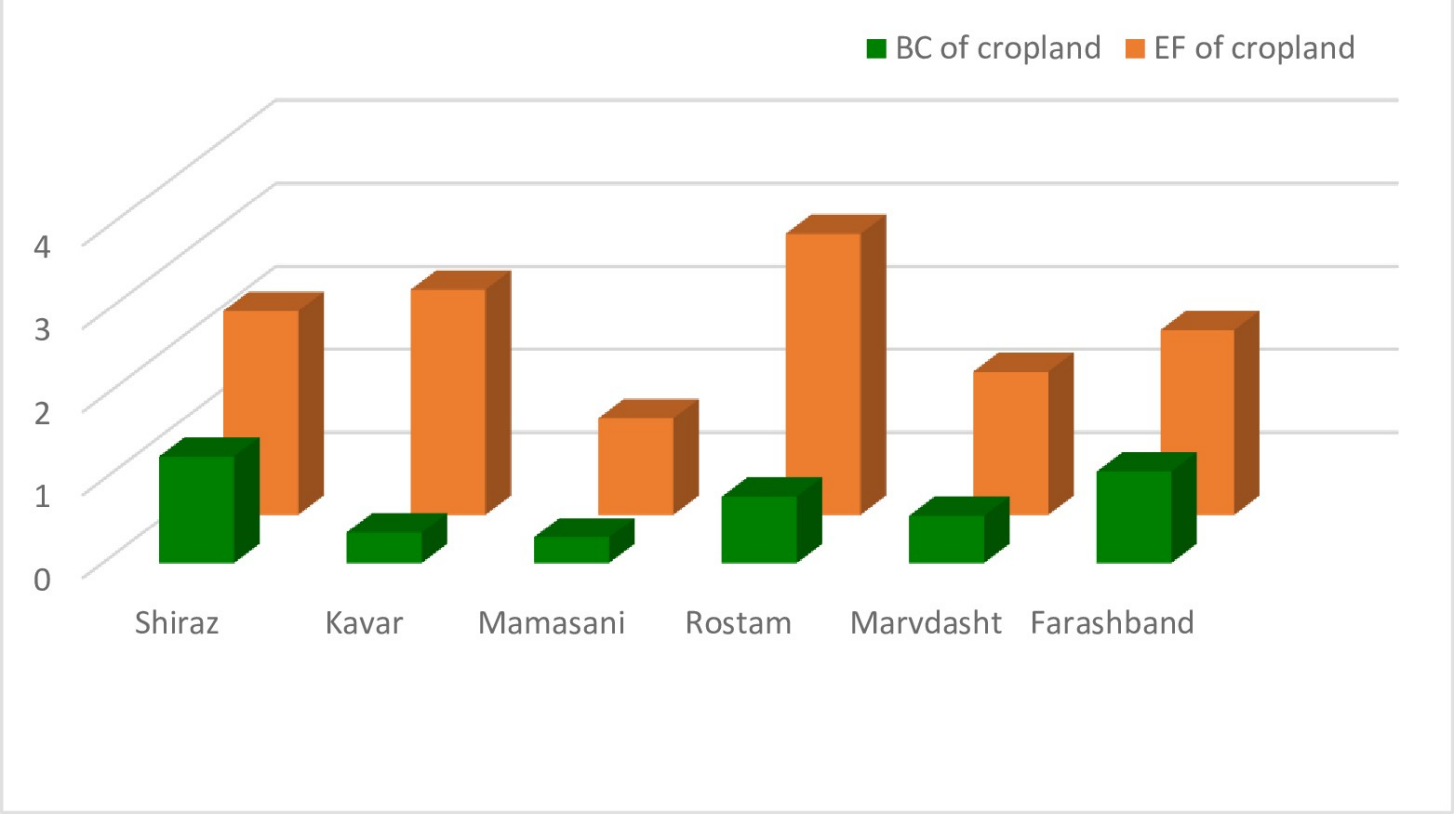
BC and EF of cropland of the counties in Fars province.

Analysis of the results of BC and EF accounting in the agricultural sector yielded findings similar to those for total indices calculation (Fig 10). The farmers`consumption of natural resources (EF) in terms of agricultural activities exceeded their BC in all of the counties. They also had severe ecological deficit with a negative trend due to the difference in ecological indices for agriculture. Indeed, the ecological index was in a low range of 0.5 to 1 in all of the counties (Table 4).

**Fig 11:**
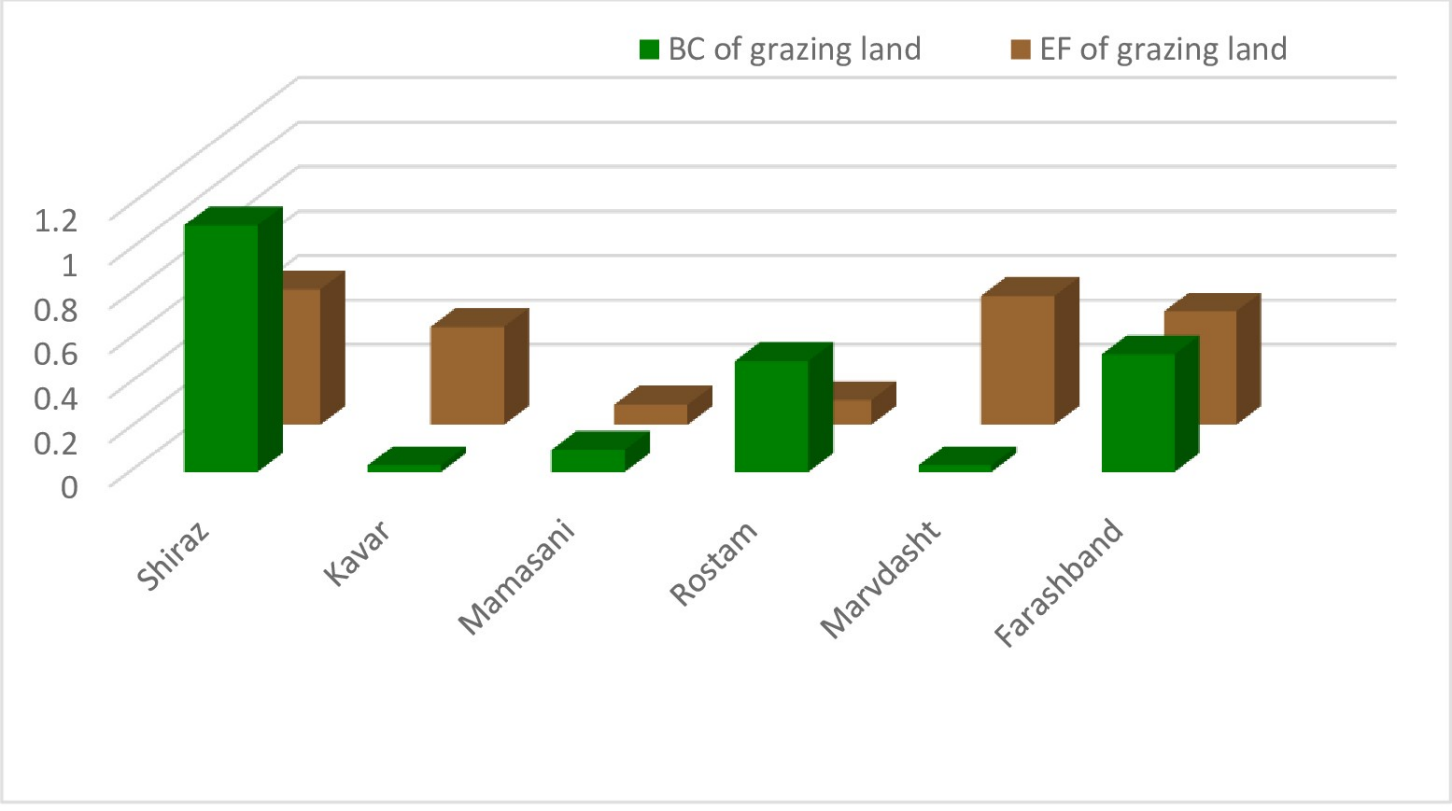
BC and EF of grazing land of the counties in Fars province.

The environmental situation of pasture at the county level was the same as shown in previous sections for the rural level and AESCs; pastures had the least acceptable condition in comparison with the total index and agriculture in terms of the society consumption of natural resources (Fig 11). Kavar and Marvdasht were two counties with ecological deficit equaling -0.41 and -0.55, respectively, indicating an unsustainable situation. The BC/EF ratio was also less than one in both counties. The other four counties had a minimum acceptable condition due to the environmental situation.

### 3.2. Sustainability comparison of villages

Ecological index (BC/EF) was known as quantitative index for measuring the sustainability, thus it has been used in this study to measure the sustainability of studied villages as well. Based on literature, BC/EF>1 shows sustainable manner with ecological reserve and BC/EF<1 showed unsustainability with ecological deficit. The results of ecological indices accounting suggest that the villages are ranked in a range of unsustainable to quite unsustainable. The villages have been categorized into three primary groups based on two main parameters, mean and standard deviation (*x*±*σ*). The first group, which has named relatively unsustainable, includes villages with a BC/EF ratio of greater than 0.7 (10 villages), the second one (unsustainable villages) includes those with an ecological index of between 0.4 and 0.7 (11 cases) and the last group (the most unsustainable) contains the villages with a ratio of less than 0.4 (29 villages). Rural grouping would provide better comparisons especially due to the social situation. The results of ANOVA, comparing the three groups are presented in Table 5.

**Table 5:** ANOVA results of comparison of triple villages groups regarding the variable research in theoretical model.

Groups	Relatively unsustainable		Unsustainable		The most unsustainable		F	Sig.
Variables	Mean	SD	Mean	SD	Mean	SD		
Village population	797.60^a^	637.57	961.73^a^	645.28	2139.97^b^	1596.85	5.74	0.006
Social participation	12.99^a^	1.90	12.04^ab^	2.14	10.93^b^	2.32	3.50	0.038
Use of green technologies	8.30^a^	2.29	6.85^ab^	2.29	6.01^b^	1.97	4.44	0.017
Extension managerial activities	8.45^a^	3.92	7.72^a^	2.41	7.31^a^	2.68	0.576	0.566
Attitude toward frontier economics	34.86^a^	7.16	37.96^a^	7.23	43.34^b^	3.14	4.58	0.015
Attitude toward eco-development	43.21^a^	9.46	42.39^a^	4.55	36.40^b^	7.43	11.59	0.0001
Attitude toward deep ecology	45.32^a^	2.36	42.46^a^	5.38	37.46^b^	6.22	8.82	0.001

• Variables scale: (Social participation: 0–24); (Green technologies: 0–14); (Extension managerial activities: 0–33); Attitude toward frontier economics, deep ecology and eco-development: 12–60).

• The means denoted with similar letters were not significantly different at the 0.05 level in the LSD test.

There was a significant difference among the population means of the three different groups of villages (sig = 0.006). The most unsustainable villages (those with the highest population mean of 2140 person) had a significant difference with the population means of the other two groups (Table 5). *Population* is a key factor of increased societal pressure on the environment and natural resources which is considered as the first component of POET model of human ecology by Duncan, as well. In terms of ecological indices, the situation of more highly populated villages (the most unsustainable) was more unsuitable and critical than the other two groups of less populated villages. Fatemi et al. [61] also indicated the negative effect of population on natural resources in terms of agricultural land conversions with great pressure of intensified cultivation on the rural lands over time.

Comparing different rural groups in terms of social participation (sig = 0.038) and the use of green technologies (sig = 0.017) indicated that there were significant differences among different groups. The means of social participation and green technologies use were the highest in the group of relatively unsustainable villages with the amounts of 12.99 and 8.30, respectively (Table 5). The means of these two variables were significantly different between the relatively unsustainable group and the other two groups. The results showed that the participation of people in the villages with a more suitable ecological index (relatively unsustainable) was greater than that of people in the other villages with lower ecological indices. This is the same for the use of green technologies; the use of these kinds of technologies was higher in the relatively unsustainable villages with an ecological index of close to one. Due to the different theories of human ecology, *technology* is another effective factors of this discipline`s model, it is also mentioned in POET model of human ecology and in accordance with the findings of ANOVA test of current study regarding significant differences of use of green technologies among diverse rural groups. According to the scale range of the variables, social participation and use of green technologies were at a moderate to low level for all of the groups. Based on the results of ecological indices accounting, most villages were in a range of unsustainability in terms of society’s pressure on the environment and natural resources. The ecological index was worse in the villages that had less participation and lower use of green technologies. The important role of eco-friendly technologies in sustainable agriculture have been demonstrated in Rezaei-Moghaddam and Karami [30] and Salehi et al. [63]. There are also the studies which were represented the improvement of environmental and natural resources conservation by social participation enhancement [64, 65].

There was no significant difference among the three rural groups in terms of extension managerial activities (sig = 0.566). The mean of this parameter was low (due to its score scale) in all of the groups and equaled 8.45, 7.72 and 7.31, respectively (Table 5). It indicated the low efficiency and effectiveness of conventional extension activities in order to reduce society pressures on the environment and natural resources. Despite the vital role of extension managerial activities as *organization* component of POET model of human ecology, but based on the ANOVA results, extension organization did not have had impressive role in sustainability achievement of studied areas, so far. Indeed, extension activities have not been successful in terms of rural societies transitioning towards a sustainable livelihood for environmental conservation, which is its most important mission. The weak efficiency of conventional extension activities of Iran had been reported in another study conducting in the rural areas of Iran [55].

According to the results of the comparison between the three rural groups in terms of paradigmatic perspectives regarding environmental management, there were significant differences among groups for the main three environmental paradigms (Table 5). The mean of attitude toward frontier economics in the most unsustainable group (43.34) was higher than the means of the other two and was significantly different (sig = 0.015). The perspective of this rural group for the other two environmental paradigms was the lowest. The means for attitude toward eco-development and deep ecology were 36.40 and 37.46, respectively (Table 5); this was lower than the mean of the other two groups and was significantly different (sig = 0.0001). The findings revealed the dominant thought of each rural group in terms of paradigmatic perspectives of environmental management. Frontier economics was the main perspective of the most unsustainable villages while the relatively unsustainable and unsustainable villages favored eco-development and deep ecology. Indeed, the most unsustainable group had a greater pressure on the environment and natural resources. In the other two groups that had a more suitable perspective for the environment there was lower pressure on resources as indicated by the calculated ecological indices.

### 3.3. Causal model analysis: Determinants of ecological index

**Fig 12:**
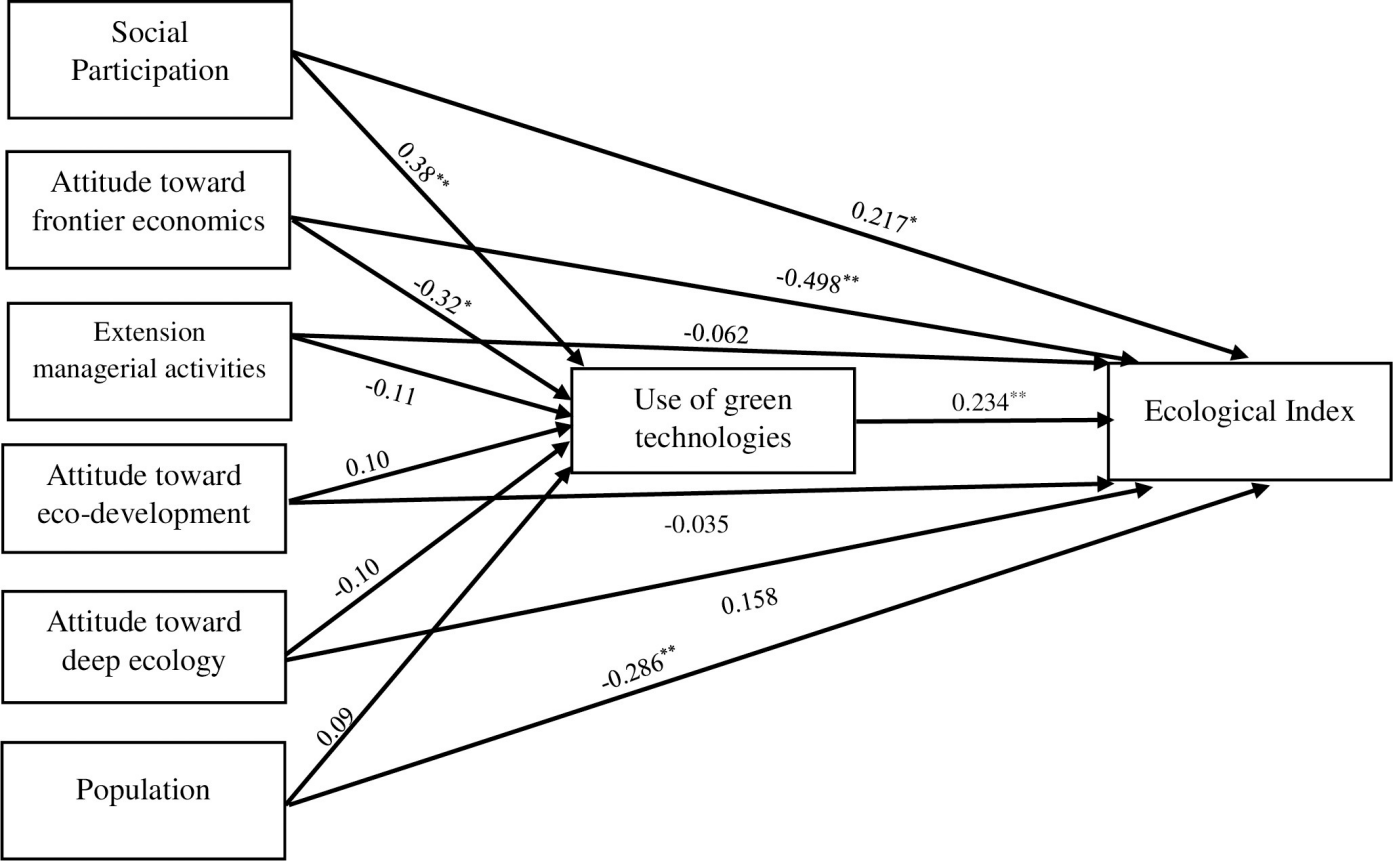
Casual model of factors influencing ecological index.

The results of path analysis of a causal model regarding factors influencing the ecological index of villages are shown in Fig 11. Before performing the model analysis, it is necessary to check the goodness of fit measures of the model. The chi-square was 41.255 and the degrees of freedom (df) equaled 15 (Probability level = 0.10). The ratio of chi-square to df was less than 5, which is one of the main conditions. NFI, CFI and GFI were calculated as less than 0.90 for this model. RMSEA was also less than 0.10. The adequate values of fit measures indicated the compatibility of the data-model. As it is shown in Fig 12, the findings of causal model were in accordance with the components of POET model of human ecology. The results of causal model were presented as follows.

#### 3.3.1. Effects on the *use of green technologies*

First, the effects of model variables on the *use of green technologies* were analyzed due to the important role of this factor as mediator variable. Social participation had a positive and significant direct effect on the use of green technologies in rural societies (β = 0.38, P = 0.003) suggesting that social participation leads to the increased application of eco-friendly technologies in rural areas. The attitude toward frontier economics had a negative and significant direct effect on the use of green and eco-friendly technologies (β = -0.32, P = 0.014). The attitude toward the paradigmatic perspective of frontier economics led to the reduction of green technologies application by farmers.

#### 3.3.2. Effects on the *ecological index*

Second, the effects of model variables on the *ecological index* were discussed. The factors were reported based on their effect intensity, respectively. The first most important factor in terms of affecting the ecological index was the dominant paradigmatic perspective of the society toward environmental management. The model demonstrated that the perspective of frontier economics had a strong negative and significant direct effect on the environment index and the pressure on natural resources (β = -0.498, P = 0.0001). If in consideration of economic issues, the dominant perspective of the society is that economic values have a higher priority than the intrinsic value of the environment, the reduction of ecological index, higher pressure and more human exploitation of the environment and natural resources would be inevitable. According to Table 6, this variable with a negative effect on the use of green technologies also had a negative indirect effect on the ecological index (β = -0.075). The effective role of attitude on the environmental conservation was mentioned and confirmed in Malek-Saeidi et al. [66] and Fatemi & Rezaei-Moghaddam [67] as well.

**Table 6:** Path results, direct and indirect effects of variables on ecological index.

Variables	Standardized total effects	Standardized direct effects	Standardized indirect effects	P
Attitude toward frontier economics	-0.574	-0.498	-0.075	**0.0001**
Village population	-0.264	-0.286	0.022	**0.007**
Use of green technologies	0.234	0.234	0.000	**0.041**
Social participation	0.307	0.217	0.089	**0.051**
Attitude toward deep ecology	0.135	0.158	-0.023	**0.174**
Extension managerial activities	-0.86	-0.062	-0.025	**0.563**
Attitude toward eco-development	0.004	-0.035	0.040	**0.770**

Population as another important variable in the model had a negative and significant direct effect on the ecological index (β = -0.286, P = 0.007). The pressure on the environment and natural resources was greater in more populated villages. The third effective variable on the ecological index was the use of green technologies based on the model (Fig 12 and Table 6). The positive direct and significant effect on the use of green technologies on the ecological index and natural resources consumption (β = 0.234, P = 0.041) indicated that the rural areas that have a higher tendency to use eco-friendly technologies in place of former traditional methods had a better environmental condition. The ecological indicator also was in a logical range for these villages in comparison with those villages that rarely use green technologies. This finding is in line with Salehi et al. [63]. Social participation as the fourth factor had a positive direct and significant effect on the ecological index (β = 0.217, P = 0.051). The rural areas with higher social participation had a more suitable condition than the other villages in which the inhabitants have less participation (Fig 12 and Table 6). Since social participation leads to transfer of environmental information among inhabitants, the importance of environmental issues and the responsibility for protection of natural resources would be growing in these societies. Social participation had also a positive indirect effect on the ecological index based on the variable of green technology use (β = 0.089). It is in line with the results of Bayona et al. [68].

### 3.4. Analysis of paradigmatic perspectives of environmental management: Agricultural policy makers of Fars province

**Fig 13:**
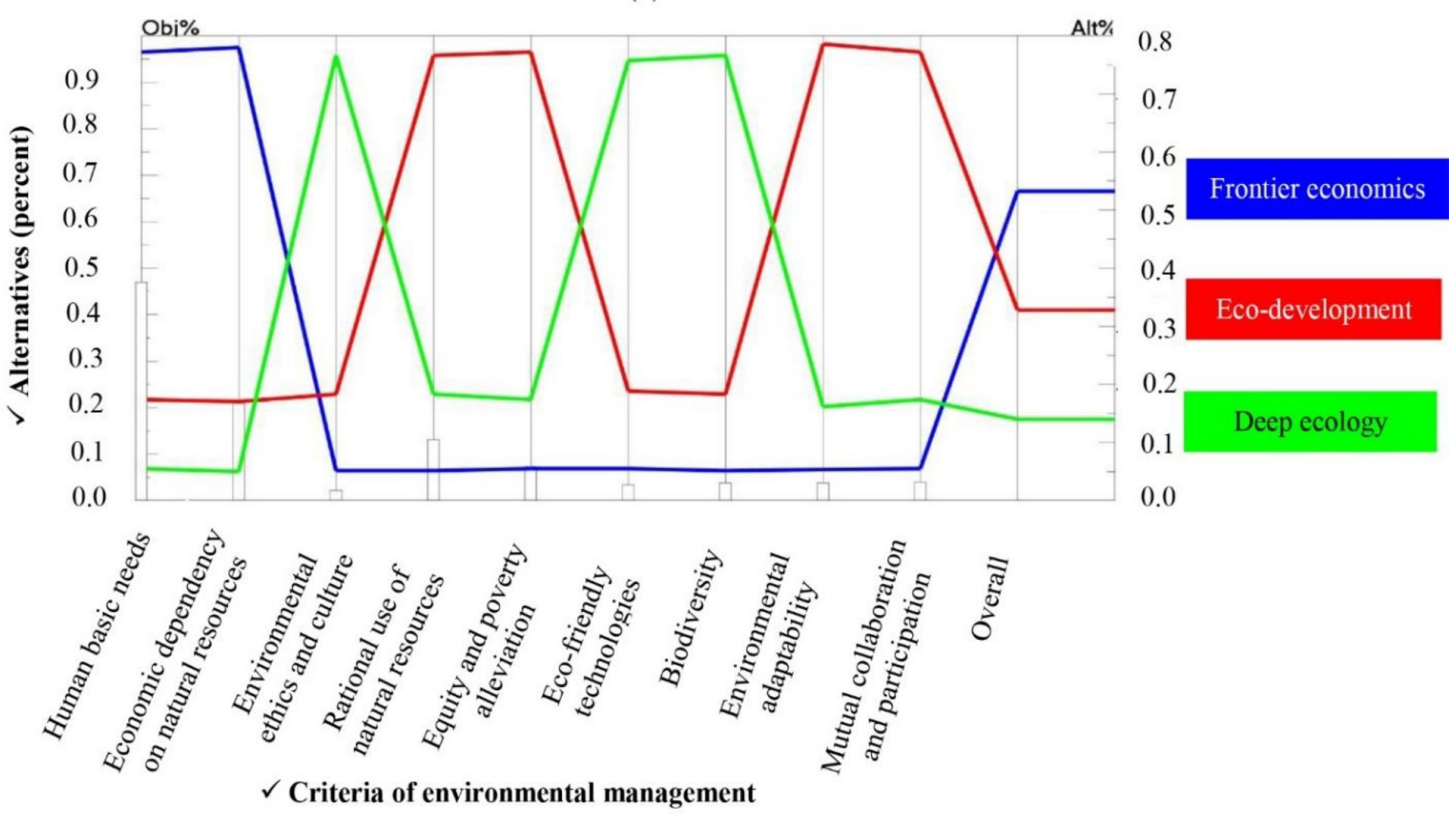
Priority of different environmental management paradigms for sustainable agriculture as perceived by AEOAJ.

**Fig 14:**
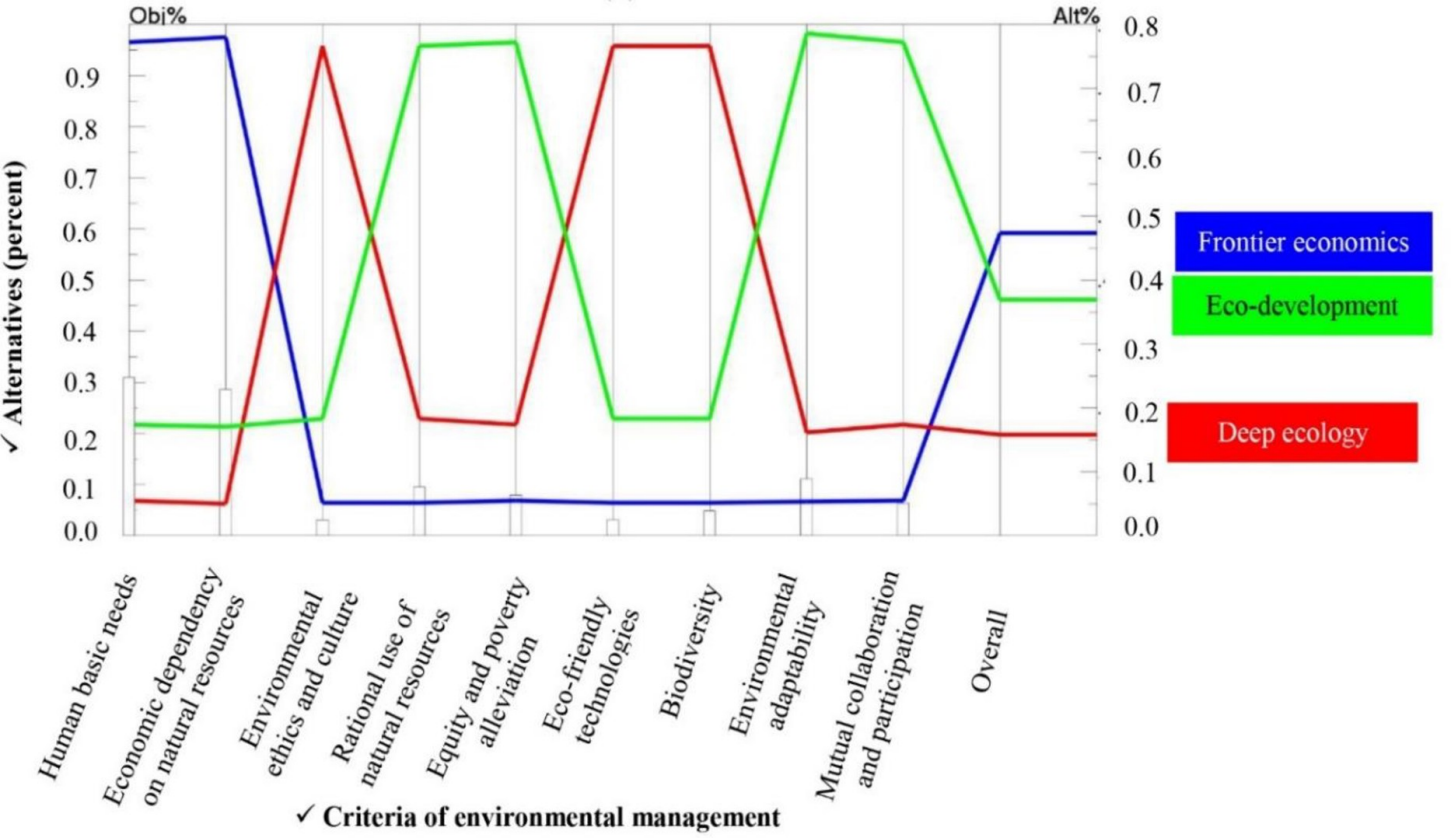
Priority of different environmental management paradigms for sustainable agriculture as perceived by AOAJ.

Organizations have a significant role in environmental studies in terms of human-nature relation. In Figs 13 and 14, the perspectives of two main managers and agricultural experts of the Organization of Agriculture Jihad of Fars province in terms of environmental management paradigms have been analyzed and compared. It would be helpful to recognize the functional base of agricultural managers and policy makers as well as agricultural extension managers of the province in terms of the ecological situation of rural areas of Fars. A measurement theory to establish the priorities of the elements of the hierarchy and the consistency of the judgmental data provided by the groups of respondents. Indeed, the priorities and the consistency was calculated in this step. Evaluation of priorities is used to compare the relative contribution of the elements in each level of the hierarchy to an element in the adjacent upper level. Synthesis of priorities was conducted to calculate a composite weight for each alternative, based on preferences derived from the comparison matrix. Following the calculation of the composite weight, the relative priority of the environmental management paradigm for sustainable agriculture was provided. The consistency in AHP is defined as the cardinal transitivity between judgments [69]. A consistency ratio provides a measure of the probability that the pairwise comparison matrix was filled in purely at random. The number 0.2 says that there is a 20% chance that the decision maker will answer the questions in random manner [70]. The inconsistency measures the logical inconsistency of judgments and is useful for identifying possible errors in judgments as well as actual inconsistencies in the judgments themselves. Overall, the consistency ratio should be less than 0.1 [71]. If the consistency ratio is greater than 0.1 then the decision makers have to re-evaluate their judgments in pairwise comparison matrix until the ratio is finally less than 0.1.

*AEOAJ of Fars province*. According to Fig 13, the top managers and authorities of agricultural extension of the province ranked the frontier economics paradigmatic perspective to achieve sustainable agricultural development as their first priority. Eco-development and deep ecology were ranked after frontier economics. The final weights of these paradigms were calculated as 0.532, 0.328 and 0.140, respectively (Table 7). According to the final weights of each paradigm, frontier economics had the highest priority by the top managers of agricultural extension of Fars province, eco-development had moderate importance and the environmental perspective of deep ecology was accorded the lowest priority with considerable difference from the other paradigms. Indeed, economic growth and implementing the strategies to maximize agricultural production are seen as the master key to all of the agricultural challenges according to the dominant paradigmatic perspectives of the top authorities of agricultural extension of Fars province.

**Table 7:** Summary of results for AHP analysis about effects of environmental management models in two managerial groups.

Alternatives	AEOAJ	AOAJ
Frontier Economics	0.532 (1)	**0.473 (1)**
Eco-development	0.328 (2)	**0.369 (2)**
Deep Ecology	0.140 (3)	**0.158 (3)**

The ranks of each alternative are presented in parentheses.

Human basic needs and economic dependency on natural resources with the weights of 0.464 and 0.203 were the two main criteria with the highest priorities based on the opinions of top managers and the agricultural extension experts of the Organization of Agriculture Jihad of the province (Table 8). These two criteria focus on economic aspects and are consistent with the frontier economics paradigm. Rational use of resources (0.125) and equity and poverty alleviation (0.067) were ranked third and fourth by this group (Table 8) and the weights of mutual collaboration and participation and environmental adaptability were 0.034 and 0.032, respectively (Table 8). Finally, biodiversity (0.030), eco-friendly technologies (0.028) and environmental ethics and culture (0.016) were considered to be least important by this group and were grouped in the last priorities (Table 8). The inconsistency ratio for this group’s pairwise comparisons was 0.09 which is less than the tolerable level of 0.1 and acceptable (Table 8).

**Table 8:** Prioritizing and ranking of the criteria for sustainable agricultural development (AEOAJ).

Components	AEOAJ
Human basic needs	**0.464 (1)**
Economic dependency on natural resources	**0.203 (2)**
Environmental ethics and culture	**0.016 (9)**
Rational use of resources	**0.125 (3)**
Equity and poverty alleviation	**0.067 (4)**
Eco-friendly technologies	**0.028 (8)**
Biodiversity	**0.030 (7)**
Environmental adaptability	**0.032 (6)**
Mutual collaboration and participation	**0.034 (5)**
Inconsistency Ratio	**0.09**

Scale: if 1 = equally important, if 3 = moderately more important, if 5 = strongly more important, if 7 = very strongly more important, if 9 = overwhelmingly more important; 2, 4, 6 and 8 are intermediate values that can be used to represent shades of judgment between the five basic assessments (The ranks of each criterion have been mentioned in the parenthesis).

Based on findings, environmental criteria such as eco-friendly technologies and biodiversity were considered to be the least important by agricultural extension top managers of Fars province. It is reasonable to see this type of weak perspective toward environmental protection by the group that is responsible for the main macro policy making and decision making of agricultural extension at the provincial level, suggesting negative environmental consequences for agriculture of the province. According to the dominant paradigmatic viewpoints of top agricultural extension authorities of the province, the intensifying trend of environmental crisis and extended natural resources degradation observed in Fars supported the results of ecological indices accounting of earlier sections and unsustainable condition of studied villages.

*AOAJ of Fars province*. Similar to findings for the key agricultural extension managers, top managers and executives of the Organization of Agriculture Jihad of Fars province selected the frontier economics paradigmatic viewpoint as the main goal to achieve sustainable agricultural development (Fig 14). The total weight of this paradigm was assessed at 0.473. Eco-development and deep ecology placed in the next priorities with weights of 0.369 and 0.158 (Table 7). These two executive groups were similar in prioritizing the frontier economics paradigm and considering deep ecology as the least important paradigm with noticeable difference. The results regarding the dominant paradigmatic perspectives of agricultural policy makers, top managers and other authorities are the same with Fatemi and Rezaei-Moghaddam [54].

Human basic needs with the weight of 0.303 was ranked first by this group (Table 9). Economic dependency on natural resources (0.280) and environmental adaptability (0.1015) were assigned to the second and third ranks, respectively. Rational use of resources, equity and poverty alleviation and mutual collaboration and participation had moderate importance in the beliefs of the AOAJ group with the respective weights of 0.090, 0.073 and 0.058 (Table 9). Finally, biodiversity (0.042), eco-friendly technologies (0.025) and environmental ethics and culture (0.023) were accorded the least priority and were placed in the last ranks by the top managers and high executives of the Organizations of Agriculture Jihad of Fars province. As shown in Table 9, the inconsistency ratio of the pairwise comparisons matrix of this group was 0.08, which is acceptable statistically.

**Table 9:** Prioritizing and ranking of the criteria for sustainable agricultural development (AOAJ).

Components	AOAJ
Human basic needs	**0.303 (1)**
Economic dependency on natural resources	**0.280 (2)**
Environmental ethics and culture	**0.023 (9)**
Rational use of resources	**0.090 (4)**
Equity and poverty alleviation	**0.073 (5)**
Eco-friendly technologies	**0.025 (8)**
Biodiversity	**0.042 (7)**
Environmental adaptability	**0.105 (3)**
Mutual collaboration and participation	**0.058 (6)**
Inconsistency Ratio	**0.08**

Scale: if 1 = equally important, if 3 = moderately more important, if 5 = strongly more important, if 7 = very strongly more important, if 9 = overwhelmingly more important; 2, 4, 6 and 8 are intermediate values that can be used to represent shades of judgment between the five basic assessments (The ranks of each criterion have been mentioned in the parenthesis).

Comparison of the two groups of top agricultural extension managers and top policy makers and authorities of the Organizations of Agricultural Jihad of the province revealed economic criteria such as human basic needs and economic dependency on natural resources to be the main criteria for agricultural development by the both groups, as these two economic criteria were placed in the first and second ranks by the AEOAJ and AOAJ groups. The environmental criteria, such as eco-friendly technologies and biodiversity, had the least priorities due to the viewpoints of these two groups. Thus, the current environmental crisis, as well as extended degradations, are not unexpected in the rural areas of the province based on the purely economic perspective of key executives of the agriculture and extension sectors.

## 4. Conclusion

The paper tried to calculate the sustainability score of different human activities. There are different indices and indicators to calculate the sustainability of different kinds of human activities including agriculture. The EF, as a quantitative index for determining the human portion of the environment and natural resources [36, 48, 51], was assessed in current study. Findings revealed that the whole studied area had not good condition as sustainability assessing through EF index. In other word, there was the great pressure on natural resources by rural people, especially in their agricultural activities, as their exploitation of resources exceeded the natural regenerative rate of resources. Continuing the current trend, would create a worsened environmental crisis and foster movement toward unsustainability.

EF/BC index was assessed at three different levels of village, agricultural service center and county in this study. Some differences could be seen if narrowing the scale. For example, there were two sustainable villages by BC/EF ratio as sustainability index. There was only one sustainable service center and there was no sustainable county. It means that we neglect the small differences by expanding our measure scale from village to the county. Consideration and application of quantitative indices such as the EF in agricultural extension and development planning can result in suitable programs in order to achieve sustainable agriculture and development at the rural level.

The necessity of macro planning at national level based on research results of ecological indices accounting are emphasized [54]. In this respect, the agricultural extension experts at different levels of provincial (Organization of Agricultural Jihad and Agricultural Extension) and local (Service Centers) groups should be familiar with the application of ecological indicators in order to promote a sustainable consumption-production cycle and educate farmers about proper consumption of natural resources. In areas with high pressure on natural resources, promotion of non-farm activities without mere dependency on natural resources is suggested in order to promote environmental protection and pressure reduction on the environment and natural resources among farmers.

The human ecology model of POET enables us to analyze the complex, reciprocal relationships among population, organizations, technology as well as the environment. Based on the SEM findings, there were seven influencing components on the ecological index at rural level including village population, social participation, attitude toward different paradigmatic perspectives of frontier economics, deep ecology and eco-development, extension managerial activities as well as green technologies use. Emphasizing rational use of natural resources and considering the BC of each region without intensified pressure on natural resources are required to move toward sustainable environmental management in agriculture. Considering the proportion of the population relative to the BC of each region is an important factor so that the existing natural resources can meet the population needs. Designing, localizing and promoting green technologies in rural areas is the other requisite factor in simultaneous achievement of agricultural production to meet the needs of rural people, without abnormal exploitation of natural resources in order to achieve environmental protection and conservation. Finally, the role of different social organizations, including governmental or private institutions as well as NGOs, in participation enhancement and volunteer activities of environmental conservation is undeniable. Consideration of these key factors in agricultural extension programs to achieve sustainable agriculture is recommended.

Probing the paradigmatic perspectives of key policy makers and top managers of the agricultural sector in Iran indicated their orientation to the economic paradigms of agricultural environmental management based on AHP results. The dominant economic perspective of agricultural extension authorities can be understood from the emphasis of agricultural programs on production increase for meeting human basic needs and economic dependency on natural resources without getting value to the environmental reserve. Therefore, their decisions, policies and planning are not based on environmental issues as well as sustainable environmental management in agriculture. Thus, a paradigm shift of agricultural policy makers and managers from their pure economic perspectives toward the integrative paradigms that consider natural resources conservation in addition to economic issues should be given high priority in order to obtain sustainable environmental management. The paradigm of eco-development that emphasizes components such as rational use of natural resources, necessity of participation and mutual collaboration, as well as eco-friendly technologies application, can simultaneously consider economic growth and environmental protection [8]. Based on the trend of this study in terms of EF accounting as an ecological indicator at the rural level and the usability of such results for local levels, it is recommended to repeat and analyze this kind of research in other rural areas of Iran as well as the other countries worldwide.

## Supporting information

S1 File (SAV)

S2 File (SAV)

## Abbreviation

EF: Ecological Footprint
BC: Biocapacity
gha: Global Hectare
AHP: Analytical Hierarchy Process
POET: Population, Organization, Environment, Technology
IPCC: Intergovernmental Panel on Climate Change
WWF: World Wide Fund for Nature
AESC: Agricultural Extension Service Centers
EFA: Ecological Footprint Accounting
GFN: Global Footprint Network
EM: Ecological Modernization
Yf: Yield Factor
EQF: Equivalence Factor
SPSS: Statistical Package for Social Sciences
AMOS: Analysis of a Moment Structures
AOAJ: Agricultural managers and experts of Organization of Agriculture Jihad
AEOAJ: Agricultural Extension of Organization of Agriculture Jihad
ANOVA: Analysis of Variance
SD: Standard Deviation
LSD: Least Significance Difference
Sig.: Significance level
df: Degree of freedom
SEM: Structural Equation Model
NFI: Normed Fit Index
CFI: Comparative Fit Index
GFI: Goodness-of-Fit Index
RMSEA: Root Mean Square Error of Approximation
